# Maternal immunization and vitamin A sufficiency impact sow primary adaptive immunity and passive protection to nursing piglets against porcine epidemic diarrhea virus infection

**DOI:** 10.3389/fimmu.2024.1397118

**Published:** 2024-05-15

**Authors:** Joshua O. Amimo, Husheem Michael, Juliet Chepngeno, Kwonil Jung, Sergei A. Raev, Francine C. Paim, Marcia V. Lee, Debasu Damtie, Anastasia N. Vlasova, Linda J. Saif

**Affiliations:** ^1^ Center for Food Animal Health, Department of Animal Sciences, College of Food, Agricultural and Environmental Sciences, Ohio Agricultural Research and Development Center (OARDC), The Ohio State University, Wooster, OH, United States; ^2^ Department of Veterinary Preventive Medicine, College of Veterinary Medicine, The Ohio State University, Columbus, OH, United States; ^3^ Department of Animal Production, Faculty of Veterinary Medicine, University of Nairobi, Nairobi, Kenya; ^4^ Department of Immunology and Molecular Biology, School of Biomedical and Laboratory Sciences, College of Medicine and Health Sciences, University of Gondar, Gondar, Ethiopia

**Keywords:** swine, gut-mammary gland-sIgA axis, passive lactogenic immunity, suckling piglets, porcine epidemic diarrhea virus (PEDV), vitamin A supplementation

## Abstract

Porcine epidemic diarrhea virus (PEDV) causes a highly contagious enteric disease with major economic losses to swine production worldwide. Due to the immaturity of the neonatal piglet immune system and given the high virulence of PEDV, improving passive lactogenic immunity is the best approach to protect suckling piglets against the lethal infection. We tested whether oral vitamin A (VA) supplementation and PEDV exposure of gestating and lactating VA-deficient (VAD) sows would enhance their primary immune responses and boost passive lactogenic protection against the PEDV challenge of their piglets. We demonstrated that PEDV inoculation of pregnant VAD sows in the third trimester provided higher levels of lactogenic protection of piglets as demonstrated by >87% survival rates of their litters compared with <10% in mock litters and that VA supplementation to VAD sows further improved the piglets’ survival rates to >98%. We observed significantly elevated PEDV IgA and IgG antibody (Ab) titers and Ab-secreting cells (ASCs) in VA-sufficient (VAS)+PEDV and VAD+VA+PEDV sows, with the latter maintaining higher Ab titers in blood prior to parturition and in blood and milk throughout lactation. The litters of VAD+VA+PEDV sows also had the highest serum PEDV-neutralizing Ab titers at piglet post-challenge days (PCD) 0 and 7, coinciding with higher PEDV IgA ASCs and Ab titers in the blood and milk of their sows, suggesting an immunomodulatory role of VA in sows. Thus, sows that delivered sufficient lactogenic immunity to their piglets provided the highest passive protection against the PEDV challenge. Maternal immunization during pregnancy (± VA) and VA sufficiency enhanced the sow primary immune responses, expression of gut–mammary gland trafficking molecules, and passive protection of their offspring. Our findings are relevant to understanding the role of VA in the Ab responses to oral attenuated vaccines that are critical for successful maternal vaccination programs against enteric infections in infants and young animals.

## 1 Introduction

Diarrheal diseases in young animals cause enormous economic losses annually to global livestock production due to high death rate, weight loss, retarded growth, treatment expenses, and trade restrictions on animals and animal source foods from diseased nations (1, 2). Porcine epidemic diarrhea (PED) is an (re)emerging disease causing serious economic losses to global swine production (3–7). Despite numerous biosecurity improvements developed by many farms for PED prevention, it remains a challenging disease to keep out of swine farms since the virus is highly transmissible and survives in the environment. Currently, many swine farms maintain seronegative status for PED virus (PEDV); however, when outbreaks occur, they suffer massive losses of neonatal piglets. Therefore, developing better strategies to protect neonatal piglets from this disease is indispensable for neonatal health and to reduce the economic impacts on swine producers.

PEDV replicates in the villous epithelial cells of the intestine (5, 8–10), causing diarrhea. Other clinical signs of PEDV include anorexia, depression, vomiting, and dehydration. These clinical signs vary depending on the previous exposure history and age of pigs (5, 8, 11–13). Owing to the high virulence of PEDV and the immaturity of the neonatal piglet immune system, targeting maternal passive lactogenic immunity is the most effective method to protect neonatal piglets against PED (14–19). Maternal lactogenic immunity through colostrum and milk offers initial protection of neonatal piglets since sows do not transfer immunoglobulins through the placenta during pregnancy (16). Studies have shown that exposing sows to mild strains of PEDV induces strong maternal lactogenic immunity capable of eliciting cross-protection against virulent PEDV (5, 18, 20). Previous fundamental studies of transmissible gastroenteritis virus (TGEV) infections in swine defined the trafficking of pathogen-specific IgA+ plasmablasts to the mammary gland (MG) and transport of secretory IgA (sIgA) antibodies (Abs) into the colostrum and milk as the gut-MG-sIgA immunological axis (14, 15, 19, 21). Reviews of these and subsequent studies confirmed and extended these findings (16, 18, 21–23). Recent studies reported that PEDV Abs in blood and MG secretions display different levels of neutralizing Abs, whereby the highest Abs titers were observed in colostrum, followed by milk, and then serum (23–25). Moreover, prior studies of TGEV (19, 21) and some studies of PEDV (18, 26) have documented a positive correlation between the levels of sIgA Abs in milk and the prevention of TGEV or PEDV deaths in suckling piglets, respectively. These results emphasize the value of lactogenic immunity in colostrum/milk for the initial passive defense of neonatal piglets against PEDV infection.

Planned exposure of sows to PEDV through the “feedback” method has been deemed to be a feasible approach of offering short-term immunity, lowering morbidity and pre-weaning deaths in swine farms (6, 8, 16, 27, 28). However, this method presents a risk of transmitting other pathogens found in the inoculum, which may trigger other disease outbreaks. On the other hand, swine producers in the USA and elsewhere have been using maternal inactivated and recombinant PEDV vaccines to protect their herds against PEDV infection (5). However, these vaccines do not provide complete protection against the disease since some vaccinated sows do not develop protective lactogenic immunity to protect their nursing piglets (5, 20). Although sow vaccination decreased mortality in young piglets, it did not reduce diarrheal severity or prevent virus shedding after inoculation, leading to the retarded growth of the affected pigs. Therefore, there is a need to develop a vaccination strategy that will prevent severe diarrhea and piglet mortality and reduce viral shedding to prevent disease transmission.

The dietary VA requirements in swine depend on the physiological condition of the animal as has been described by the National Research Council (29). Puls, in 1994, published reference values for serum VA in each age group of swine, including suckling, nursery piglets, growers, and adult pigs (400–500 ng/mL) and lactating sows (250–400 ng/mL) (30). A recent survey of VA and mineral levels in conventional swine by Greiner and colleagues reported serum VA levels of 20–280 ng/mL in suckling piglets and 30–320 ng/mL in lactating sow (31). Same authors reported hepatic VA levels for suckling piglets and lactating sows as 18–63 and 80–530 µg/g, respectively. Vitamin A deficiency (VAD) is a leading micronutrient deficiency and is mostly found in children under 5 years of age and animals. Triggers of VAD include infectious diseases, low VA in foods consumed, reduction in feed intake, reduced absorption, inability to mobilize VA from the liver, and accelerated depletion of VA via increased excretion. Imbalances in immunity, retinoid metabolism, and tissue repair responses may interact or synergize to increase the severity of infections in VAD individuals. VA induces profound immune modulation and helps to maintain epithelial integrity, thereby contributing to reduced disease. Retinoic acid (RA) is the immunologically active metabolite of VA with multiple effects on the immune system (32, 33). VA enhances immunity through increasing IgA Ab responses, increasing trafficking of B and T cells to the gut or other mucosal tissues, increasing lymphocyte proliferation responses to antigens, preventing apoptosis, and preserving and repairing cells lining the mucosal surfaces (34–36). These findings have emphasized a need to monitor and often increase VA levels to optimize the efficacy of vaccination programs. Here we assess if PEDV infection and VA supplementation to VAD sows during gestation and lactation reduces the severity of PEDV and prevents virus shedding in suckling piglets by potentiating passive lactogenic Ab responses.

To mitigate PED, swine producers require an efficacious strategy that stimulates a strong immune response without threat of disease. Thus, an inexpensive supplement/vaccine adjuvant such as VA that boosts maternal immunity from a previous PEDV natural infection could help in providing better passive lactogenic protection to nursing piglets (26, 37, 38). To this end, in-depth knowledge of Ab-mediated protection mechanisms against PEDV infection is critical for the development of a more effective PEDV vaccine. In this study, we hypothesized that oral VA supplementation to VAD multiparous sows previously infected with PEDV would boost lactogenic immunity, thereby enhancing neonatal piglet immune protection against PEDV infection and disease. Here we evaluated primary maternal lactogenic immunity following PEDV exposure in multiparous sows and passive protection of their suckling piglets; additionally, we assessed whether VA supplementation of VAD sows would enhance these immune responses.

## 2 Materials and methods

### 2.1 Virus

The sows and piglets were inoculated with 1 × 10^5^ plaque-forming units (PFU) of wild-type PEDV (PC22A strain) diluted in Minimal Essential Media (Life Technologies, Carlsbad, CA, USA). The PEDV inoculum was prepared as described previously by Langel et al. (23, 26). The PEDV PC22A strain was isolated and cultured in Vero cells as described previously (39, 40). Cell culture-adapted PEDV PC22A was used in the PEDV-specific Ab assays.

### 2.2 Experimental design

Multiparous (parity 2–5) PEDV naïve/uninoculated sows were assigned to VAD or VA-sufficient (VAS) diets at gestation day (GD) ~30 (the gestation period of sows is 114 days). The VAS groups received a regular commercial sow diet with recommended VA content (1,200 IU/lb of feed). The VAD+VA group received a premix with no VA content from GD30 to the end of the experiment. Additionally, one group of VAD sows (VAD+VA) was supplemented with oral VA, retinyl palmitate (30,000IU) daily in feed starting at GD ~76 until the end of experiment. The parity distribution of sows included VAS-mock (3× parity 4, parity 5), VAS+PEDV (2× parity 3, parities 4 and 5), VAD-mock (parity 3, 2× parity 4, parity 5), VAD+PEDV (parities 2, 3, 4, and 5), VAD+VA-mock (2× parity 3, 2× parity 4), and VAD+VA+PEDV (2× parity 2, parities 4 and 5). The sows and piglets were inoculated with 1 × 10^5^ PFU dose of wild-type PEDV PC22A strain, while the control/mock sows were inoculated with sterile MEM without virus. The sows were inoculated orally at GD ~90, and all piglets were challenged with PEDV at 3–5 postpartum days (DPP). Sow feces were collected every other day for 2 weeks starting from post-inoculation day 0 (PID0), while piglet fecal samples were collected daily from piglet post-challenge day 0 (PCD0) to PCD7, followed by every other day until PCD14. Diarrhea scores were determined based on fecal consistency as 0 to 3 for solid, pasty, semi-liquid, and liquid, respectively. Any score ≥2 was considered as diarrhea (23, 37, 41). Blood samples were collected from sows for immunological assays at GD 83–93, GD 109–115, DPP 3–5, DPP 10–12, and DPP 18–21 (euthanasia), while mammary gland (MG) biopsies were collected from sows at three time points: GD 83–93, GD 109–115, and DPP 3–5. Milk samples were collected from sows at four time points after farrowing: DPP 0 (colostrum), DPP 3–5, DPP 10–12, and DPP 18–21 (euthanasia). The piglets were weighed at birth (DPP 0), DPP 3–5, 7–8, 10–12, and 17–21. The blood from piglets for serum processing was collected three times at DPP 3–5 (PCD 0), DPP 10–12 (PCD 6–8), and DPP 18–21 (PCD 12–16). The defined euthanasia end points for sows and piglets were DPP 18–21. However, the piglets that were moribund and could not reach the end of the experiment were removed based on the following criteria (1): inability to ambulate, hence not able suckle and/or drink water for 2 consecutive days (2), a loss of 10%–20% of body weight within 3–5 days and/or clinical dehydration, and (3) inability to stand and/or unconsciousness with no response to external stimuli. The animals were euthanized by first administering intramuscularly a premix of telazol/ketamine/xylazine at a dose of 4.4/2.2/2.2 mg/kg body weight for complete sedation, followed by an intravenous injection of Fatal plus (pentobarbital, 390 mg/mL) at a dose of 86.6 mg/kg body weight. At euthanasia, blood, milk, spleen, ileum, MG, and mesenteric lymph node tissues were obtained from sows for the isolation of mononuclear cells (MNC). Additionally, large and small intestinal contents were collected from all the sows and piglets at euthanasia and suspended in 5 mL MEM with protease inhibitors and centrifuged at 2,100 × *g* at 4°C for 10 min, and the supernatants were stored at -20°C until use.

### 2.3 Biological sample collection

Blood was collected aseptically from the jugular vein of the individual animals. Sow blood for MNC isolation was collected with 30% anticoagulant (acid citrate dextrose, ACD), while whole blood was collected from sows (15–20 mL) and piglets (3–5 mL) to obtain serum. The serum was obtained by centrifugation (2,100 × *g*) at RT for 20 min, and the supernatant was stored at -20°C until use.

The sows were injected with 2 mL of oxytocin (40 IU) intramuscularly to enable the collection of milk/colostrum. Milk/colostrum was filtered (70-μm cell strainer) to remove the debris. Thereafter, ~4-mL aliquots of whole milk were stored in dark tubes at -80°C for future VA testing. The rest of the milk samples were used to isolate MNCs and for the processing of whey for subsequent immunological assays.

Fecal samples for PEDV RT-PCR testing were collected from the rectum of individual sows and piglets using two sterile cotton swabs immediately prior to and after PEDV inoculation as described in the study design section. The swabs were suspended in 4 mL MEM with antibiotic–antimycotic, followed by centrifugation (2,100 × *g*) at 4°C for 10 min. The supernatants were stored at -20°C until use.

Spleen, ileum, mesenteric lymph node (MLN), and MG tissues from sows were obtained at euthanasia and placed in media (RPMI 1640 with 10 mM HEPES, 200 g of gentamicin per milliliter, and 20 g of ampicillin per milliliter; Life Technologies, Carlsbad, CA, USA). The tissues were processed to obtain MNCs for immunological assays.

### 2.4 Serum and hepatic VA concentrations

Serum from sows at different time points (GD ~30, GD ~76, GD ~90, GD ~109, and DPP 18–21), serum of piglets at euthanasia (DPP 18–21), and liver of sows and piglets (DPP 18–21) were used to measure VA concentrations using high-performance liquid chromatography (HPLC) performed at the Veterinary Diagnostic Laboratory, Iowa State University.

### 2.5 Quantification of PEDV RNA shedding by RT-qPCR

Viral RNA extraction from fecal suspensions was conducted using MagMAX™ Viral RNA Isolation Kit (Applied Biosystems, Foster City, CA, USA) and a KingFisher® 96 magnetic particle processor (Thermo-Fisher Scientific) following the manufacturer’s instructions. Viral titer was measured by TaqMan RT-qPCR using the one-step RT-PCR Kit (Qiagen, Valencia, CA, USA) as described previously (9). The limit of detection was set as previously determined, which was 10 copies/20 μL of reaction, which corresponded to 4.8 log_10_ viral RNA copies/mL of original fecal samples (9, 23).

### 2.6 Mononuclear cell isolation from tissues

The blood, spleen, MLN, MG, and ileal lamina propria were processed to obtain MNCs using previously established protocols (26, 37). Isolated MNCs were resuspended in enriched RPMI [RPMI medium containing 8% fetal bovine serum, 2 mM l-glutamine, 1 mM sodium pyruvate, 0.1 mM non-essential amino acids, 20 mM HEPES (*N*-2-hydroxy- ethylpiperazine-*N*−2-ethanesulfonic acid), and 1% antibiotic-antimycotic; Life Technologies, Carlsbad, CA, USA]. The MNC quantity and viability were examined by trypan blue exclusion and measured by using Cellometer (Nexcelom, Lawrence, MA, USA). MNCs were used for immunological assays immediately or frozen (freezing media containing 10% DMSO and 90% FBS) in liquid nitrogen until use.

Isolation of MNCs in MG secretions: The milk/colostrum samples were diluted 1:3 in sterile Dulbecco’s PBS (DPBS; pH 7.2, without calcium and magnesium) to improve the cell harvest by the dispersion of fat micelles. However, before dilution, a separate portion of whole milk/colostrum (~50 mL) was used to obtain skim milk for whey processing. The diluted and undiluted milk/colostrum were centrifuged at 800 × *g* at RT for 10 min for the separation of cell pellet, skim milk, and fat. The skim milk from the undiluted tube was retained and used to obtain whey. The cell pellet was processed to obtain MNCs as described by Chepngeno and coauthors (37, 38).

### 2.7 PEDV whole-virus Ab ELISA assay

The cell culture-adapted PC22A PEDV-infected Vero cell lysates or mock-inoculated Vero cells were used as a virus or control antigen for whole viral Ab ELISA, respectively, following protocols as described previously (23). PEDV IgG and IgA antibody ELISA was done as described previously (23). The positive control serum used in our assay was obtained from a known PEDV positive sow, while the negative control serum was obtained from a known PEDV seronegative sow. PEDV IgG and IgA Ab titers were expressed as the reciprocal of the highest dilution that had a corrected A_450_ value greater than the cutoff value (mean + three standard deviations of negative control samples).

### 2.8 PEDV-specific antibody-secreting cell (ELISPOT) assay

The Vero cells infected with PEDV and mock antigens were grown and fixed in 96-well plates as described previously for TGEV ASCs (26, 42, 43). The ELISPOT assay for the quantification of PEDV-specific ASCs was performed following previous protocols (23, 37, 38). Average spot counts were expressed per 5 × 10^5^ MNCs.

### 2.9 PEDV neutralization assay

We conducted fluorescent focus neutralization (FFN) and plaque reduction virus neutralization assays (PRVN) to measure VN Ab titers in sow/piglets serum and whey, respectively, as described previously (23, 37, 44). VN Ab titers were calculated and expressed as the reciprocal of the highest dilution of each sample showing 80% reduction in the number of plaques compared with the negative control sample.

### 2.10 Flow cytometry assays to assess immune responses

Flow cytometry staining for the evaluation of frequencies of T/B lymphocytes was done as described previously (23, 45). To assess the frequencies of B cell subpopulations, we used anti-porcine CD21-PE (clone BB6–11C9.6, Southern Biotech) and anti-porcine CD2 (clone MSA4, King Fisher Biotech) monoclonal Abs (mAbs) to stain the MNCs, followed by intracellular staining with porcine cross-reactive anti-mouse CD79β-FITC (clone AT1072, Bio-Rad) mAb. We also separately stained MNCs with mouse anti-pig IgA mAb (K61 1B4, Bio-Rad) to examine the frequencies of IgA-producing B cells among the MNCs. To assess the expression of gut homing markers (α4β7/CCR10), the MNCs were stained with porcine cross-reactive human Integrin alpha 4 beta 7/LPAM-1 (Clone # Hu117, R&D Systems) and anti-mouse CCR10 (clone 248918, R&D Systems), respectively. To evaluate the frequencies of T lymphocyte subpopulations, we stained the MNCs with anti-porcine CD4 (clone 74–12-4, Southern Biotech), anti-porcine CD8α (clone 76–2-11, Southern Biotech), and anti-porcine CD3 (clone PPT3, Southern Biotech) Abs. Additionally, PEDV-specific IFN-γ producing T cells were determined by staining the CD3^+^ MNCs with anti-porcine IFN-γ−PE Ab (clone P2G10; BD Biosciences). The T regulatory cell subpopulations were determined by using porcine cross-reactive rat anti-mouse Foxp3-FITC (clone FJK-16s; eBioscience, San Diego, CA, USA) and anti-porcine CD25 (Clone K231.3B2; Serotec, Raleigh, NC, USA) Abs. Appropriate isotype control and secondary Abs were included in each assay (23, 37). The MNCs were stained with mouse anti-porcine CD4-PE (BD Biosciences, cat. #559586), mouse anti-porcine CD8-PE-cy7 (BD Biosciences, cat. #559584), anti-porcine CD25-APC (Bio-Rad, cat. #MCA1736GA), and anti-porcine Foxp3-FITC (Invitrogen, cat. #11–5773-82) Abs to estimate the frequencies of different Treg populations (activated, inducible, and natural) among the CD4^+^ and CD8^+^ T lymphocytes. We used Accuri C6 flow cytometer (BD Biosciences, San Jose, CA, USA) with acquisition set at 50,000 events to analyze the stained samples.

### 2.11 Immunohistochemical and immunofluorescent staining of mammary gland tissues

MG biopsy samples were collected using disposable core biopsy instrument kits and placed in neutral buffered formalin (10%) for fixing at three different time points: gestation day (GD) 84–93, GD 109–115, and 3–5 days postpartum. Immunofluorescence (IF) or immunohistochemistry (IHC) was performed on fixed tissues for the detection of IgA^+^ and IgG^+^ B cells and MAdCAM-1, CCL25, or pIgR antigen. For the IHC of IgA^+^ and IgG^+^ cells, goat anti-pig IgA polyclonal Ab and goat anti-pig IgG (H+L) polyclonal Ab, respectively, were all diluted 1:100 in PBS. Both Abs were conjugated with horseradish peroxidase (Bio-Rad and Seracare, respectively). The numbers of IgA^+^ or IgG^+^ B cells were counted in stained MG sections per microscopic area at a magnification of ×200 to obtain mean numbers. For IF of MAdCAM-1, CCL25, or pIgR antigen, mAbs against human MAdCAM-1 or CCL25 (Novus Biologicals) and a polyclonal rabbit Ab against human pIgR (Proteintech) were all diluted 1:100 in PBS. The IF-stained tissues were processed by using NIH ImageJ software. The ImageJ-processed, IF-positive areas (green) were transformed into red color. Mean red-colored areas (%) positive for MAdCAM-1, CCL25, or pIgR antigen were measured in stained MG sections per microscopic area at a magnification of ×200 using NIH ImageJ software.

### 2.12 Data analysis

Means and standard errors (SE) for VN Ab titer, PEDV IgA and IgG ASCs and titers, PEDV fecal shedding, and percent body weight change were estimated for each treatment group using GraphPad Prism 5 (GraphPad Software, Inc., CA, USA). For each piglet, the body weight change was calculated relative to the weight at birth (normalized weight). The mean frequencies of different cell subpopulations were analyzed by one-way or two-way analysis of variance (ANOVA), followed by Tukey–Kramer test to determine significant differences (*p* ≤ 0.05) among the groups.

## 3 Results

### 3.1 Vitamin A supplementation rescued VAD in pregnant and lactating sows

**Figure 1:**
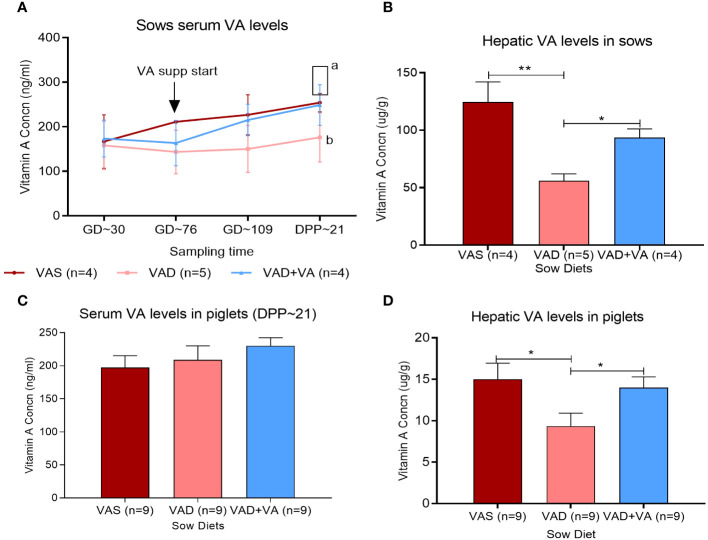
Vitamin A (VA) concentrations in serum (ng/mL) and liver (µg/g) of sows **(A, B)** fed VA-sufficient (VAS), VA-deficient (VAD), and VA-deficient with VA supplementation (VAD+VA) diets during gestation (GD) and post-parturition (DPP) and their piglets **(C, D)**. Normal range, serum (UPLC)—lactating sows: 250–400 ng/mL, nursing piglets: 400–500 ng/mL; liver (UPLC)—adult pigs: 57–286 µg/g, suckling piglets: 36–57µg/g (Veterinary Diagnostic Laboratory, Iowa State University). **(A)** Different letters denote significant differences in groups; error bars denote SE (*p < 0.05, **p < 0.01).

We induced VAD in a conventional pig model by feeding the gestating sows with a VA-deficient diet starting from 30 days of pregnancy (**Figure 1A**). The deficiency was rescued by VA supplementation of the VAD sows from gestation day 76 until the end of the experiment. We observed that oral VA supplementation to VAD sows resulted in a significant (*p* < 0.05) increase in circulating VA concentrations as observed at the end of the experiment (DPP ~21) (**Figure 1A**). Similarly, there was a relative increase in the hepatic VA concentration in VAD sows that received VA supplementation (**Figure 1B**). The analyses of serum and hepatic VA levels in the piglets showed lower mean values than the minimum levels recommended in piglets of similar age. It is worth noting that from our previous studies (37, 45), we observed that an enteric virus (rotavirus) infection resulted in a significant decrease in hepatic vitamin A levels in piglets. Hence, PEDV being more virulent than rotavirus, we believe that it directly and indirectly affects the vitamin A levels in piglets. This likely resulted from the decreased absorption of the dietary VA and increased demand for RA (needed for immune activation) and therefore increased catabolism of the stored VA. Additionally, PEDV-infected piglets have reduced appetite and do not receive adequate levels of most macro- and micronutrients. However, comparing the mean VA levels among the groups, there was no significant difference (*p* < 0.05) in the circulating VA concentration in piglets (21 days old) of the different sow groups (**Figure 1C**), but an analysis of the VA concentrations in piglet liver showed that VA supplementation of VAD sows during gestation and lactation led to significant (*p* < 0.05) increases in hepatic VA reserves in their piglets compared to VAD litters (**Figure 1D**).

### 3.2 Prior exposure to PEDV during gestation and maternal VA sufficiency enhanced the lactogenic Ab protection of nursing piglets

**Figure 2:**
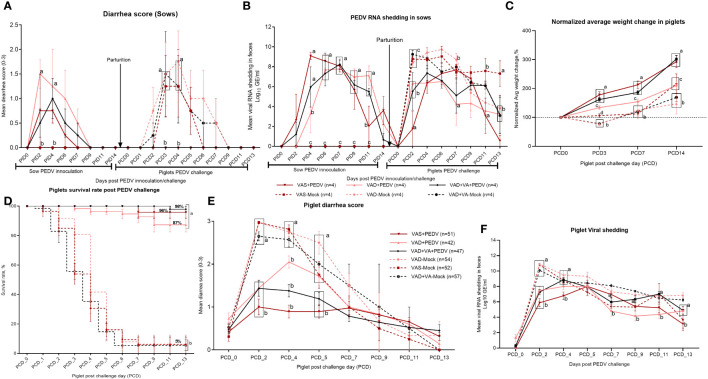
Diarrhea was determined by fecal consistency scores (0, normal; 1, pasty; 2, semi-liquid; 3, watery) in sows **(A)** and piglets **(E)**. PEDV RNA shedding was determined by qRT-PCR and expressed as log_10_ GE/mL in sows **(B)** and piglets **(F)**. Normalized average weight gain (lb) of VAS (*n* = 52), VAD (*n* = 54), VAS+PEDV (*n* = 51), VAD+PEDV (*n* = 42), and VAD+VA+PEDV (*n* = 47) litters from birth to 21 days old. Weight gain was normalized based on the piglet’s birth weight **(C)**. Survival rate **(D)** of piglets at 0–13 PEDV post-challenge days (PCD0–13); error bars denote SE. Different letters denote significant differences in groups; *p* < 0.05.

Clinical signs were variable in PEDV-inoculated sows, with some animals developing signs such as decreased or complete loss of appetite, vomiting, and/or soft-to-pasty feces. Fecal consistency and PEDV RNA shedding were recorded daily after PEDV or MEM (mock) inoculation of the sows, where we observed that none of the sows developed acute diarrhea (**Figure 2A**), although VAD+PEDV sows, but not the VAS+PEDV or VAD+VA+PEDV sow, developed soft pasty feces after PEDV inoculation at PID 2–6 (**Figure 2A**). The fecal RNA shedding results revealed a delayed onset of viral shedding in all the sows that were inoculated with PEDV during gestation compared to mock sows when their piglets were challenged (DPP 3–5) with PEDV (**Figure 2B**). All the mock sow groups had higher diarrhea scores after the piglet challenge (PCD 2–4) compared to the PEDV ± VA sows after inoculation during pregnancy (PID 2–4). Previously PEDV-inoculated sows (PEDV ± VA) did not develop diarrhea after the piglet PEDV challenge. PEDV RNA shedding titers were higher in mock sows compared with PEDV sows in the post-piglet-challenge period. These results suggest the protective and immunomodulatory roles of VA.

Piglets developed mild to severe clinical signs depending on the PEDV and dietary status of their mothers. The disease was characterized by severe diarrhea, vomiting, complete loss of appetite, and loss of body weight (**Figures 2C, E**). Mortality was most pronounced in the litters born to mock (VAS/VAD) sows (**Figure 2D**). However, we observed milder disease in the litters of PEDV-inoculated sows irrespective of the diet status, with litters of VAS+PEDV and VAD+VA+PEDV sows showing no or less clinical signs post-PEDV-challenge compared with VAD+PEDV litters (**Figures 2C, E**). The clinical parameters of the piglets after PEDV challenge (DPP 3–5) to the end of the experiment (DPP 18–21) revealed that PEDV exposure and VA supplementation to pregnant multiparous sows in the third trimester improved the lactogenic protection to their nursing piglets as evident in the higher body weight gain, increased survival rates, and reduced mean fecal consistency scores of the VAS+PED and VAD+VA+PEDV litter post-PEDV -challenge (**Figures 2C–E**). These data further suggest that VA-mediated immunomodulation may improve the piglets’ gut barrier and absorptive and digestive functions as evidenced by reduced diarrhea (**Figure 2E**) despite high viral shedding at PCD 2–6 (**Figure 2F**). Finally, we demonstrated that PEDV inoculation of pregnant sows (irrespective of diet) in the third trimester provided high lactogenic protection to piglets as shown by the significantly increased (>85%) survival rates of their litters compared to mock (~5%) and that VA supplementation to VAD sows further improved the survival rates to ~98% (**Figure 2D**). All clinical parameters (diarrhea scores, shedding, and mortality) were significantly decreased in the VAS+PEDV and VAD+VA+PEDV litters with a steady weight gain throughout the experiment.

### 3.3 VA sufficiency and PEDV exposure of pregnant sows enhanced primary maternal and lactogenic antibody responses in sows and piglet passive protection to PEDV challenge

To understand the impact of VAD, VA supplementation, and PEDV exposure during gestation and lactation of multiparous sows on primary lactogenic immune responses and passively acquired protection of their piglets, we analyzed adaptive immune responses and immune cell parameters in the blood, MG secretions, intestinal contents, and selected tissues in the sows and in the blood and intestinal contents of the piglets.

#### 3.3.1 PEDV-specific IgA and IgG ASCs

**Figure 3:**
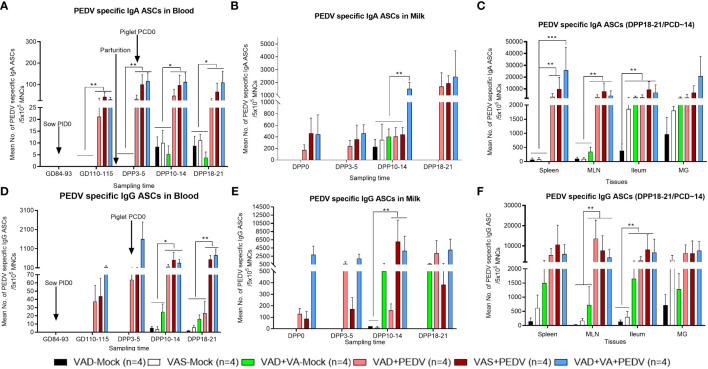
Mean numbers of PEDV-specific Ab-secreting cells (IgA/IgG ASCs) in the blood **(A, D)**, colostrum/milk **(B, E)**, and tissues **(C, F)** of mock and PEDV-infected sows fed VAS, VAD, and VAD+VA-supplemented diets during gestation and postpartum (*p < 0.05; **p < 0.01, ***p < 0.001). Error bars denote SE. (NB: Milk data at DPP18–21 for the mock group is missing since the sows ceased lactating after losing all the piglets.).

Our results demonstrated that the mean numbers of circulating PEDV IgA and IgG ASCs in the blood, milk, and selected tissues were significantly higher in the PEDV-inoculated sow groups compared to the mock groups (**Figure 3A**). Additionally, we observed numerically elevated PEDV IgA ASCs in the blood and milk of VAS+PEDV and VAD+VA+PEDV sows compared with mock (VAS and VAD) and VAD+PEDV sows during gestation and lactation (**Figures 3A, B, D, E**). We further observed that VAD+VA+PEDV sows maintained numerically higher mean numbers of PEDV-specific IgA ASCs in blood (**Figure 3A**) and milk (**Figure 3B**) prior to parturition and, along with VAS+PEDV sows, throughout lactation, especially late lactation. Moreover, VAS+PEDV and VAD+VA+PEDV sows had high and mostly comparable mean numbers of PEDV IgA and IgG ASCs among the tissues analyzed at 18–21 days post-parturition (**Figures 3C, F**). Interestingly, although the VAD+PEDV sows maintained somewhat lower mean numbers of PEDV IgA ASCs in blood, milk, and tissues during gestation and lactation, especially by DPP 18–21, compared with the other two PEDV groups, their piglets had improved lactogenic protection (87% survival rate) and mean body weight gains compared to the mock litters (5% survival rate) as shown in **Figures 2C, D**.

#### 3.3.2 PEDV-specific IgA and IgG Ab titers

**Figure 4:**
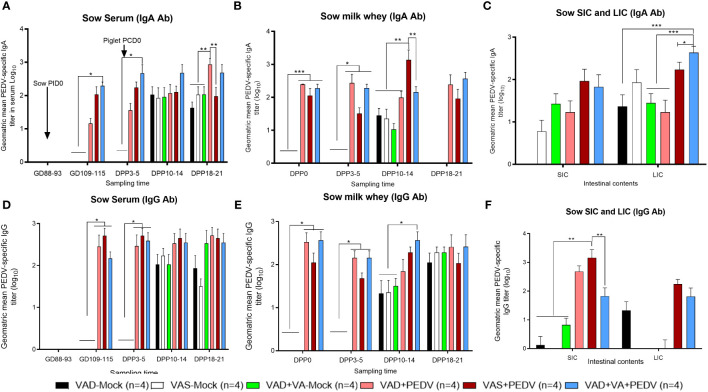
Mean PEDV-specific IgA Ab titers **(A–C)** and IgG Ab titers **(D–F)** in the serum, milk, and intestinal contents (SIC/LIC) of PEDV/mock-inoculated sows fed VAS and VAD (± VA) diets during gestation and lactation (*p < 0.05; **p < 0.01, ***p < 0.001). SIC, small intestinal contents; LIC, large intestinal contents; error bars denote SE. (NB: Milk data at DPP18–21 for the mock group is missing since the sows ceased lactating after losing all the piglets.).

Our data demonstrated that the PEDV IgA Ab titers in serum were comparable in VAS+PEDV and VAD+VA+PEDV sow groups prior to parturition (GD 109–115) and in early lactation (DPP 3–5). These groups also maintained significantly higher Ab titers prior to parturition and 3–5 days post-parturition compared to VAD+PEDV and mock groups (**Figures 4A, D**), consistent with the PEDV ASCs results. No significant differences were seen in PEDV-specific IgA and IgG Ab titers in the colostrum (PPD 0) of PEDV pre-exposed sows irrespective of VA diet, although VAD+VA+PEDV and VAD+PEDV sows had slightly elevated PEDV IgA and IgG Ab titers in milk in early lactation compared to the other groups (**Figures 4B, E**). Additionally, the PEDV-inoculated sows (irrespective of diet) maintained numerically elevated IgA and IgG Ab titers during mid lactation compared to the mock groups. An analysis of the PEDV Ab titers in the intestinal contents of the sows showed that the PEDV IgA Ab titers in the small intestinal contents (SIC) did not vary significantly among the groups. However, in the large intestinal contents, these Abs were significantly elevated in VAD+VA+PEDV groups compared to VAS+PEDV, VAD+PEDV, and mock sows (**Figure 4C**). The VAS+ PEDV group had significantly elevated levels of PEDV IgG-specific Abs in SIC compared with the mock groups and the VAS+VA+PEDV sows (**Figure 4F**).

#### 3.3.3 VA sufficiency and PEDV exposure of pregnant sows enhanced PEDV-specific IgA and IgG Ab titers in piglets

**Figure 5:**
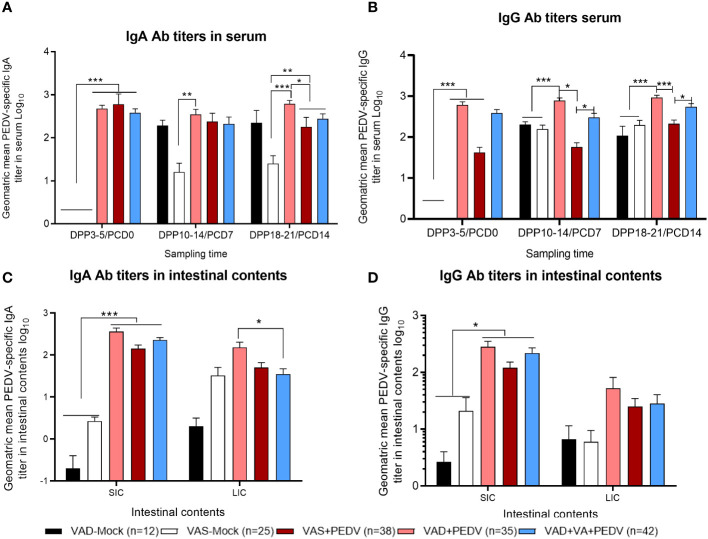
Mean PEDV-specific IgA Ab titers **(A, C)** and IgG antibody titers **(B, D)** in the serum (upper) and intestinal contents (lower) of piglets of PEDV/mock-inoculated sows fed VAS and VAD (± VA) diets during gestation and lactation (*p < 0.05; **p < 0.01; ***p < 0.001). SIC, small intestinal contents; LIC, large intestinal contents; error bars denote SE. (NB: Data at DPP18–21 for the VAD+VA-mock group litters are missing since the sows lost all the piglets.).

When we assessed the PEDV-specific Ab titers in piglets’ serum and intestinal contents, the PEDV IgA Ab titers were slightly higher in the serum of VAS+PEDV litters prior to the PEDV challenge (PCD0) compared to the VAD+PEDV and VAD+VA+PEDV litters and significantly higher than in mock litters (**Figure 5A**). Interestingly, the VAD+PEDV litters showed significantly higher PEDV IgA and IgG serum Ab titers at PCD7 and PCD14 vs. the other groups (**Figures 5A, B**). The PEDV IgA and IgG Ab titers in the intestinal contents (SIC and LIC) were comparable in previously PEDV-exposed sow litters. The PEDV IgA Ab titers were significantly higher in the SIC of these groups than in the mock litters (**Figures 5C, D**). The piglets of VAD+PEDV sows had significantly higher PEDV IgA Ab titers in LIC compared with the VAD+VA+PEDV group.

#### 3.3.4 PEDV challenge (± VA) of piglets increases PEDV VN Ab titers in sow and piglet serum and maintains VN Ab titers in milk throughout the experiment

**Figure 6:**
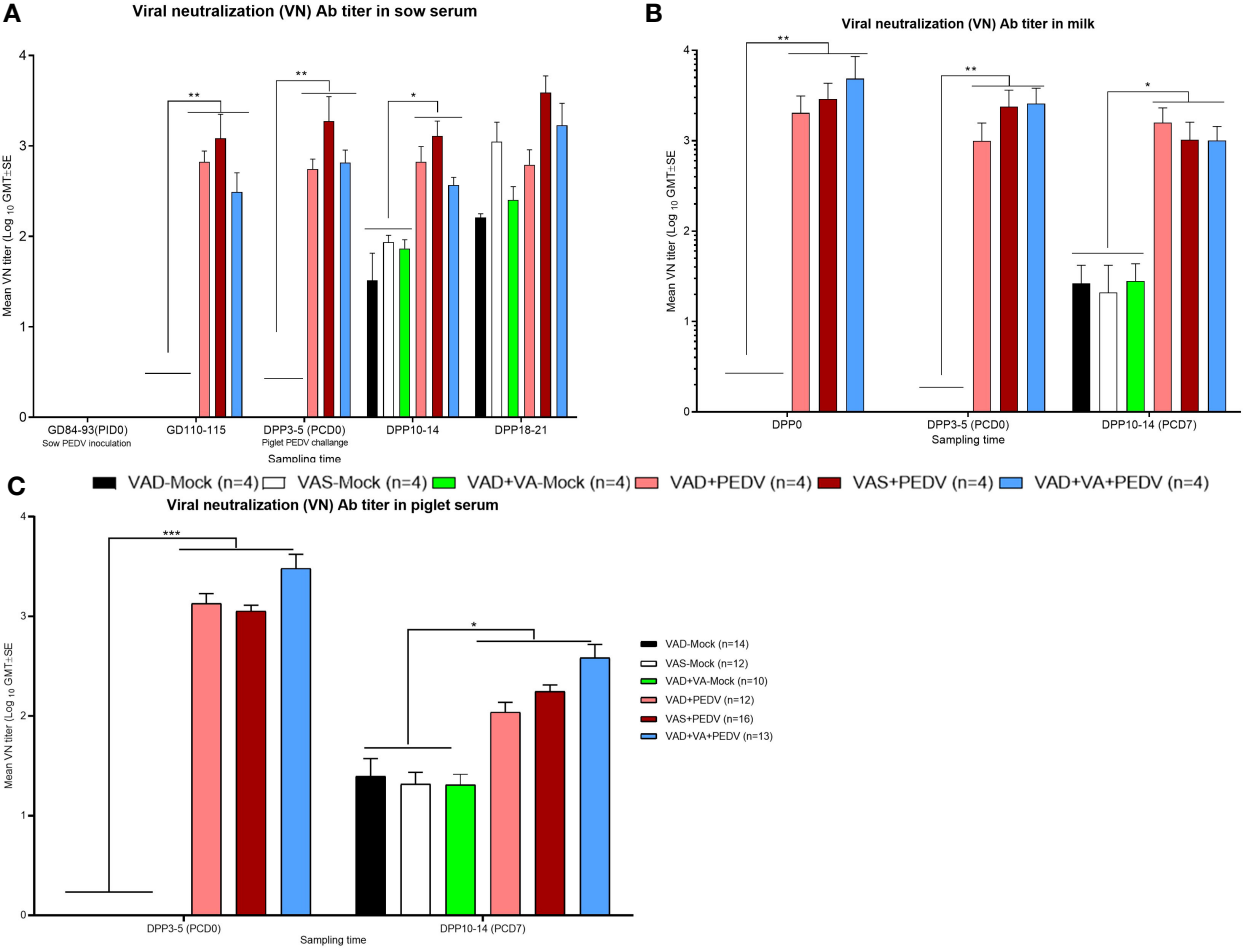
Mean PEDV-neutralizing (VN) Abs in the blood (**A**, sows; **C**, piglets) and milk **(B)** of mock/PEDV-inoculated sows fed VAS, VAD, and VAD+VAsupplemented diets during gestation and postpartum and their piglets. Error bars denote SE. *p < 0.05; **p < 0.01; ***p < 0.001.

PEDV-specific virus neutralization (VN) Ab titers: Consistent with our ELISA Ab titer results, the PEDV VN titers were significantly higher in the serum and milk of previously PEDV-exposed sow groups prior to (GD ~110) and after parturition (DPP 3–5 and DPP 10–14) compared with the mock groups (**Figure 6A**). Within the previously PEDV-exposed groups, the PEDV VN Ab titers were numerically higher in the blood of VAS+PEDV sows compared to VAD+PEDV and VAD+VA+PEDV sows during gestation and lactation. We observed that PEDV-inoculated sows (± VA) had significantly higher levels of PEDV VN Ab titers in serum and milk after the PEDV piglet challenge (DPP 10–12/PCD 7) compared with the mock counterparts (**Figure 6**). We did not observe major differences in VN Ab titers in serum and milk (except in milk at DPP 0 and 3–5) between VA-supplemented and non-supplemented VAD sows during gestation and after parturition. The PEDV VN Ab titers were significantly higher (*p* < 0.001) in the serum of piglets of PEDV-inoculated sows compared to those of mock sows before (PCD 0) and after the PEDV challenge (PCD 7), indicative of PEDV Abs acquired through colostrum (**Figure 6C**). Importantly, the litters of VAD+VA+PEDV sows had the highest PEDV VN Ab titers numerically at PCD 0 and 7 (**Figure 6C**), coinciding with higher levels of PEDV-specific IgA ASCs observed in the blood and milk of their mothers prior to parturition and throughout lactation (**Figures 3A, B**), suggesting a role of VA in regulating immune responses.

### 3.4 PEDV exposure and VA sufficiency increases the frequencies of Ig-secreting B cells, IgA^+^ B cells, and gut homing (α4β7^+^) cells in blood, milk, and tissues

To investigate the effects of VA supplementation and PEDV exposure in pregnant sows on the distribution of different B cell phenotypes in systemic and mucosal MNCs, we stained the isolated MNCs with pan B cell CD79β Ab, followed by staining with CD2 and CD21 Abs. We evaluated the frequencies of Ig-secreting (CD79β^+^CD2^+^CD21^-^), naïve (CD79β^+^CD2^+^CD21^+^), activated (CD79β^+^CD2^-^CD21^+^), and resting/memory (CD79β^+^CD2^-^CD21^-^) B cell subpopulations (**Supplementary Tables S1A–C**). Our results showed that VAD+VA+PEDV sows had an elevated mean frequency of Ig-secreting B cells (normalized to GD ~90, prior to PEDV inoculation) in blood prior to parturition and maintained higher levels in blood and milk throughout lactation compared to the other groups (**Supplementary Tables S1A, B**). An analysis of the mean frequencies of Ig-secreting B cell subpopulations in tissues revealed no clear trends between the groups; however, there were trends toward higher levels of Ig-secreting B cells in MG in all the sows, irrespective of their diet and PEDV exposure status, compared to other tissues (**Supplementary Table S1C**). Similarly, no clear trends were observed in the frequencies of activated, naïve, and memory B cells in blood, milk, and tissues; however, VAD+PEDV sows had relatively elevated activated B cells in MLN and ileum. The VAD+VA+PEDV sows had the highest frequencies (although not significantly higher) of naive B cell subpopulations in blood and milk at DPP 3–5 and MLN (**Supplementary Table S1C**).

**Figure 7:**
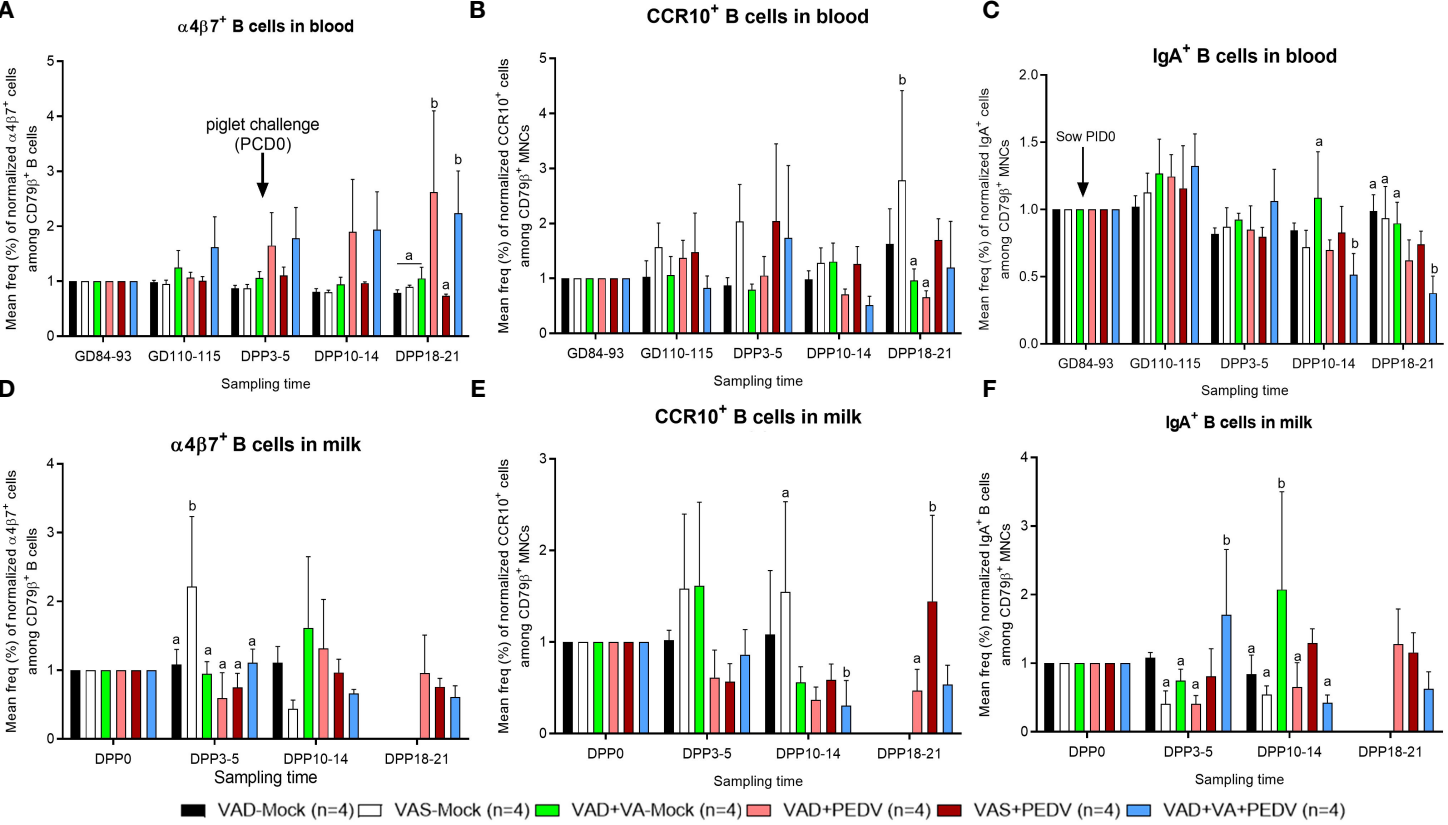
Mean frequencies (%) of normalized IgA+ (left), α4β7^+^ (middle) and CCR10^+^ (right) cell subpopulations among B lymphocytes (CD79β^+^) in the blood **(A–C)** and milk **(D–F)** of PEDV/mock-inoculated sows fed VAS and VAD (± VA) diets during gestation and postpartum. Frequencies were normalized to GD~90, prior to PEDV inoculation; error bars denote SE. (NB: Milk data at DPP18–21 for the mock group is missing since the sows ceased lactating after losing all the piglets.). Different alphabetical letters (a, b) indicate significant differences (p<0.05) at the same time point among treatment groups.

An analysis of gut homing (α4β7^+^ and CCR10^+^) cell subpopulations among the B lymphocytes in blood (normalized to GD ~90, prior to PEDV inoculation) revealed that VAD+VA+PEDV sows had numerically elevated α4β7^+^ integrin B cell subpopulations pre-parturition and during lactation compared to the other groups (**Figure 7A**). Interestingly, we observed relatively elevated frequencies of α4β7^+^ cells in the blood of VAD+PEDV sows postpartum, which were comparable to the levels in VAD+VA+PEDV sows, and significantly elevated frequencies in both groups compared with the other groups at DPP 18–21. An analysis of CCR10^+^ cells among the B lymphocytes did not reveal any significant trends in the frequencies of these cell subpopulations between the treatment groups; however, VAS (± PEDV) and VAD+VA+PEDV sows had marginally higher levels of CCR10^+^ cells in blood at DPP 3–5 (**Figure 7B**). No marked trends were observed in the frequencies of α4β7^+^ and CCR10^+^ in milk among the groups (**Figures 7D, E**), however, VAS-Mock sows had significantly elevated frequencies of α4β7^+^ cells at DPP3-5 and CCR10^+^ at DPP10-14. We examined the frequencies of IgA^+^ cells among the B lymphocytes and the proportion of IgA^+^ cells that express α4β7^+^ integrin (IgA^+^ α4β7^+^) in blood, milk, and tissues (**Figures 7C, F**; **Supplementary Table S1**). VAS+PEDV and VAD+VA+PEDV sows showed slightly elevated IgA^+^ B cells in blood prior to parturition (GD 110–115) and early in lactation (DPP 3–5), but they were significantly elevated in the mock sows at DPP 18–21 compared with the VAD+PEDV groups (**Figure 7C**). All groups had higher frequencies of IgA^+^ B cells in their colostrum (**Figure 7F**), and the frequencies decreased during lactation in all sow groups, except for sows in the VAD+VA (± PEDV) groups which had an increase in the frequencies of these cells at DPP 10–14 and DPP 18–21, suggesting a possible role of VA in IgA^+^ B cell migration to MG after PEDV infection. Among different tissues, VAD+PEDV sows had the highest IgA^+^ and IgA^+^ α4β7^+^ B cell frequencies in their ileum and MG MNCs compared to the other groups (**Supplementary Table S1D**).

### 3.5 VA supplementation enhanced the frequencies of CD4^+^ T cells in milk and IFN-γ-producing CD4^+^ cells in blood and induced regulatory T cells in tissues

**Figure 8:**
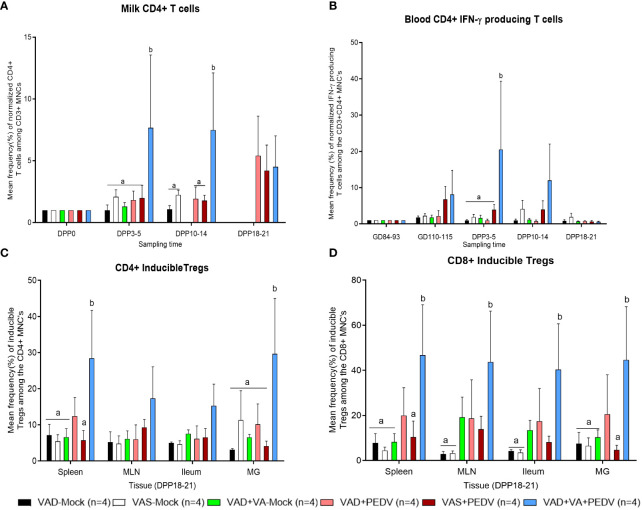
Mean frequencies of normalized CD4^+^ T cells in milk **(A)**. **(B)** Mean frequencies of normalized PEDV-specific IFN-γ producing CD4^+^ T cells in the blood of different sow groups. Frequencies in blood were normalized to GD~90, prior to PEDV inoculation; in milk to colostrum (DPP0). **(C)** Mean frequencies of CD4+ inducible Tregs (CD4^+^Foxp3^+^CD25^-^) and **(D)** Mean frequencies of CD8^+^ inducible Tregs (CD8^+^Foxp3^+^CD25^-^) in various tissues at euthanasia (DPP18–21) of PEDV/mock-exposed sows fed on VAS, VAD, and VAD+VA diets during gestation and lactation period. Error bars denote SE. Different alphabetical letters (a, b) indicate significant differences (p<0.05) at the same time point among treatment groups

To investigate the influence of VAD, VA supplementation, and PEDV exposure on the adaptive T cell immune responses, we evaluated the frequencies of helper and cytotoxic T cells among the CD3^+^ lymphocytes in the MNCs of systemic and mucosal tissues and MG secretions of sows (**Supplementary Figure S1**). Additionally, we evaluated the frequencies of the IFN-γ producing and regulatory CD4^+^/CD8^+^ T cell populations among the CD3^+^ T cells (**Supplementary Figures S2**-**S5**). Although there were no significant differences in the frequencies of CD4^+^ T cells in blood, milk, and tissues among the groups, we observed numerically higher frequencies of these cells in mock groups, irrespective of their diet, compared to their PEDV-inoculated counterparts (**Supplementary Figures S1A, C, D**). When we normalized the frequencies of CD4^+^ T cells in milk based on the frequencies in colostrum (DPP 0), we observed that there was a relatively gradual increase in the frequencies of these cells in the VAD+VA+PEDV group throughout lactation (**Figure 8A**) compared to the other groups. The frequencies (%) of CD8^+^ T cells were also comparable among the groups at all the time points in blood (**Supplementary Figure S1B**), while the frequencies of these cells were increased in the milk of VAD+VA-mock at early lactation and in VAD+VA+PEDV at late lactation (**Supplementary Figure S1D**). There were no significant differences in the frequencies of these cells in tissues examined at euthanasia, although the VAS+PEDV group had relatively higher frequencies of these cells in spleen and MLN, while the frequencies were higher in VAD+VA-mock sows in ileum and MG (**Supplementary Figure S1F**).

The frequencies (%) of IFN-γ producing CD4^+^ T cells were elevated in blood MNCs of the VAD+VA+PEDV and VAS+PEDV groups prior to parturition and in early lactation compared to the other groups (**Figure 8B**; **Supplementary Figure S2A**). On the other hand, the frequencies (%) of IFN-γ-producing CD8^+^ T cells were higher in the blood of VAS (± PEDV) at GD 110–115 and DPP 10–14 and in VAD+VA+PEDV at DPP 3–5 (**Supplementary Figure S2B**). In milk, the frequencies (%) of IFN-γ producing CD4^+^/CD8^+^ T cells were higher in VAD+VA-mock colostrum (DPP 0) and DPP 3–5 (**Supplementary Figures S2C, D**). We observed that VAS+PEDV sows maintained relatively high frequencies of these cells throughout lactation (**Supplementary Figures S2C, D**). Upon examining the tissues, the frequencies (%) of IFN-γ producing CD4^+^ T cells were higher in VAD-mock (± PEDV) in spleen, MLN, ileum, and MG (**Supplementary Figure S2E**), while IFN-γ-producing CD8^+^ T cells were relatively higher in the VAD+VA-mock group in all tissues (**Supplementary Figure S2F**).

There were no clear differences in the frequencies of CD4^+^/CD8^+^-activated and natural Treg cells in blood and milk among the groups. However, mock groups, irrespective of diet, appeared to have higher frequencies of CD4^+^/CD8^+^-activated Tregs in blood throughout the experiment (**Supplementary Figures S3A, D**). The frequencies of CD4^+^/CD8^+^ iTregs were significantly (*p* < 0.05) higher in the blood of VAD+VA+PEDV and VAD+PEDV groups throughout the experiment (**Supplementary Figures S3B, E**). In milk, the frequencies of CD4^+^ iTregs were elevated in the colostrum of all groups, except in the VAD+PEDV group. The frequencies of these cells gradually decreased in all groups by DPP 3–5 before peaking in late lactation (**Supplementary Figures S4B, E**). At euthanasia (DPP 18–21), the frequencies of CD4^+^/CD8^+^ iTregs were elevated in the VAD+VA+PEDV group compared with the other groups among all the tissues (**Figures 8C, D**; **Supplementary Figure S5B, E**).

### 3.6 Increased numbers of IgA^+^ and IgG^+^ cells and expression of pIgR, MAdCAM-1, and CCL25 in MG postpartum

The IgA^+^ and IgG^+^ cells were mostly found in intra- or interlobular stroma of stained MG tissue, visualized as spherical/oval shape resembling plasma cells or plasmablasts. On the other hand, MAdCAM-1 and CCL25 were expressed on the endothelial cells lining the intralobular or interlobular vessels (**Supplementary Figures S6**, **S7**) We observed a moderate to large amount of MAdCAM-1 or CCL25 in the endothelial cells lining the variable-caliber vessels present in the interstitium of the MG. pIgR expression was observed on the epithelial cells lining the alveoli of MG (**Supplementary Figure S8**), where a moderate to large amount of pIgR was detected in the epithelial cells lining the acini. The frequencies of IgA^+^ and IgG^+^ cells as well as MAdCAM-1 and CCL25 expression in the MG were similar among all sows and were slightly elevated in all sows after parturition (**Supplementary Figures S9A–D**). Our results showed that all sow groups maintained similar levels of IgA+ cells in MG throughout the experiment, except VAD+PEDV sows that had the highest level post-parturition (**Supplementary Figure S9A**). Surprisingly, MAdCAM-1 expression in MG postpartum was increased in VAD-mock and VAD+PEDV sows, suggestive of a possible VA compensatory mechanism in these groups; however, further studies are needed to clarify the effects of VAD on MAdCAM-1 expression. Although there were no significant differences in mean MG areas positive for pIgR among groups at all time points, we observed a trend toward higher pIgR expression in MG of sows postpartum (DPP 3–5) compared with prepartum (GD 84–93 and GD 110–115).

## 4 Discussion

In this study, we evaluated the primary immune responses of multiparous pregnant sows inoculated with PEDV in the third trimester and assessed the passive lactogenic protection of their nursing piglets against PEDV infection. Previous studies have shown that inactivated or subunit vaccines have lower immunogenicity, requiring alternative delivery approaches and including adjuvants (immunostimulatory) to trigger greater innate and adaptive immunity (46). Animal research has provided evidence that optimal VA levels are needed to achieve efficient Ab response to many pathogens and that VA deficiency during infection negatively impacts innate and adaptive immunity (26, 37, 38, 47). Thus, we further assessed whether VA supplementation to VAD pregnant sows enhances these immune responses. Our results showed that the sows previously inoculated with PEDV protect their piglets (>95% survival rate) compared to noninfected/mock sows (~5% survival rate) and that VA supplementation to VAD+PEDV sows further improved the protection of their litters (~98% survival rate). Consistent with previous studies (23, 48, 49), we observed milder disease in the litters of sows previously exposed to PEDV, with litters of VAS+PEDV and VAD+VA+PEDV sows showing less or no clinical signs of the disease post-PEDV-challenge, respectively, compared with VAD+PEDV and mock litters, which had moderate to severe disease characterized by increased morbidity and mortality. The increased PEDV-specific IgG and IgA Abs and ASCs responses observed in VAS+PEDV and VAD+VA+PEDV sows, which resulted in better protection of their litters, were in agreement with the findings of previous studies (23, 26, 49–51)—for instance, de Arriba and colleagues, using a conventional pig model, demonstrated a correlation between IgA and IgG Abs [in gastrointestinal associated lymphoid tissues (GALT) and blood] and piglet protection against PEDV infection (52). Previous studies have shown the important role of VA in boosting and regulating host immunity and cellular transport between the gut and MG (26, 37, 38).

Increased IgA^+^ and α4β7^+^ B cell frequencies (in gestation/lactation) in VAD+VA+PEDV sows coincided with improved protection of their piglets against PEDV challenge as evident in reduced diarrhea and increased PEDV-specific IgA and IgG ASCs in sows and Ab responses in their piglets. Previous studies have provided evidence that VA directs immune tolerance in the body through the collective interactions of GALT, sIgA, bacterial communities, and DCs (53–55). Similar to our findings, Penkert and colleagues observed increased pneumococcus-specific Abs in VAD mice supplemented with VA at the time of pneumococcal vaccination, with no mortality in mice that received VA supplementation at immunization (56). More recently, using a conventional pig model, our lab established that VA supplementation of PEDV-exposed pregnant gilts in the third trimester enhanced primary maternal and lactogenic Ab responses and the passive protection of the suckling piglets (26). However, compared to our previous findings in gilts (primiparous) who were not maintained on VAD diets, our current study showed that multiparous VAS+PEDV sows had increased humoral immune responses (Ab and B cell responses) compared with gilts (VAS+PEDV) as evident in the increased survival of their piglets from 5.8% in VAS-mock to 96%, compared with gilts 5.7% VAS-mock to 55.9% litters. Moreover, even VAD+PEDV and VAD+VA+PEDV sows had higher piglet survival rates (of 87% and 98%, respectively) than VAS+PEDV gilts. Previous studies of humans and mice demonstrated that VA supplementation significantly increased the milk sIgA levels in postpartum women (57) and IgA ASCs in the MG of mice (58). However, using a mouse model of asthma, Schuster and colleagues observed an increase in disease severity in VA-supplemented mice compared with VAD mice, which they associated with a decrease in Th2 cell cytokines (59).

We observed a trend toward increased frequencies of various B cell phenotypes, especially Ig secreting B cells, in the blood and milk of VAD+PEDV (± VA) vs. VAS+PEDV sows at various time points during gestation and lactation. Although previous animal studies provided evidence that an optimal VA concentration is needed for effective Ab response to many pathogens, other studies, as reviewed by Ross (60), have shown that VAD animals are able to produce a robust Ab response to some antigens; hence, these responses are dysregulated by VAD, rather than merely compromised. Thus, it implies that when VAD individuals are supplemented with VA, the mechanisms for proficient immune responses are intact, even though dysregulated, and they can be restored by supplemental VA to rapidly regenerate normal adaptive immune responses (61, 62). Additionally, VAD animals tend to mobilize hepatic VA reserves to maintain adequate levels of VA in the circulation as compensatory mechanisms, although this may lead to the depletion of hepatic VA reserves in chronic VAD conditions. Kelleher and his colleague reported that long-term VAD alters retinol metabolism in lactating rats without decreasing the levels of VA in the circulation, and they attributed it to the increased cellular retinol binding protein (CRBP) expression in the liver (63). CRBP expression is regulated by dietary proteins rather than VA, and CRBP is involved in retinol accumulation and metabolism within the cells (64). Similarly, we did not observe significant differences in circulating VA levels in sows, except at euthanasia (DPP 18–21), and piglets, irrespective of their diet; however, there were significant differences (*p* < 0.05) in hepatic VA levels among the VAS, VAD, and VAD+VA groups, whereby VAD sows and their litters had lower concentrations compared with VAS and VAD+VA sows and their litters.

Numerous studies have shown that T cell immunocompetence can be affected by VAD both in terms of T cell functions and counts (65–67). Moreover, RA has been shown to induce the differentiation of Tregs and maintain their stability and immunoregulatory role (41, 68–70). Inducible Treg cells (iTreg) have been shown to play critical roles in mucosal immune tolerance and control of severe chronic allergic inflammation. In our study, CD4^+^/CD8^+^ iTregs were higher in the blood of VAD+VA+PEDV and VAD+PEDV groups throughout the experiment. The increase in these cells may be a compensatory mechanism to autoimmune processes like elevated IL17 cells. An increase in iTregs accompanied by a decrease in IL17 production has been shown to shift the balance of these cells toward effective immune homeostasis (71). Penny and colleagues demonstrated that VA affects the reprogramming of iTregs into Th17 cells during intestinal inflammation in mice (65). Surprisingly, we observed that the VAD-mock sows had elevated levels of helper (CD4^+^) and cytotoxic (CD8^+^) T cells in blood MNCs and maintained these high frequencies throughout the experiment. This was similar to the finding in beef cattle where dietary VA restriction in beef cattle enhances T cell responses crucial for controlling bovine leukemia virus (BLV), whereby increased CD4^+^ T cells contributed to a more efficient killing of BLV-infected lymphocytes, especially in secondary lymphoid organs like spleen (72).

In conclusion, our study demonstrated that maternal VAD resulted in piglet VAD which led to reduced protective B cell and Ab responses and passive lactogenic immunity to PEDV. However, prior exposure of pregnant VAD sows to PEDV significantly decreased mortality, diarrheal severity, and virus shedding following PEDV challenge in their nursing piglets compared with the mock controls, but these disease parameters were much reduced in the PEDV+VA-sufficient groups (litters of VAS+PEDV and VAD+VA+PEDV sows). Thus, we further showed that enhancing the maternal immunity of VAD sows via daily VA supplementation (immune-stimulatory adjuvant) during pregnancy (third trimester) and lactation improved the function of the gut–MG–sIgA immunological axis and enhanced the passive lactogenic immunity in nursing piglets. Our findings are important for understanding the immunomodulatory role of VA during infection/vaccination, which is critical for effective immunization programs and may lead to development of more efficacious maternal vaccines for animals and humans.

## Data availability statement

The original contributions presented in the study are included in the article/**Supplementary Material**. Further inquiries can be directed to the corresponding authors.

## Ethics statement

The animal studies were approved by Institutional Animal Care and Use Committee at The Ohio State University under the IACUC protocol# 2015A00000071. The studies were conducted in accordance with the local legislation and institutional requirements. Written informed consent was obtained from the owners for the participation of their animals in this study.

## Author contributions

JA: Data curation, Formal analysis, Methodology, Writing – original draft, Writing – review & editing. HM: Data curation, Formal analysis, Methodology, Writing – review & editing. JC: Data curation, Formal analysis, Methodology, Writing – review & editing. KJ: Data curation, Formal analysis, Methodology, Writing – review & editing. SR: Data curation, Formal analysis, Methodology, Writing – review & editing. FC: Data curation, Methodology, Writing – review & editing. ML: Formal analysis, Methodology, Writing – review & editing. DD: Data curation, Methodology, Writing – review & editing. AV: Conceptualization, Formal analysis, Funding acquisition, Supervision, Writing – review & editing. LS: Conceptualization, Funding acquisition, Supervision, Writing – review & editing.

## Supplementary material

Supplementary Figure 1 Frequencies (%) of CD3^+^CD4^+^ **(A, C, E)** and CD3^+^CD8^+^ **(B, D, F)** cells in the blood, milk, and tissues of PEDV/mock-exposed sows fed VAS, VAD, and VAD+VA diets. MLN, mesenteric lymph nodes; MG, mammary gland. NB: Milk data at DPP18–21 for the mock group is missing since the sows ceased lactating after losing all the piglets. Error bars denote SE.

Supplementary Figure 2 Frequencies (%) of IFN-γ producing cells among the CD3^+^CD4^+^ **(A, C, E)** and CD3^+^CD8^+^ **(B, D, F)** T cells in the blood, milk, and tissues of PEDV/mock-exposed sows fed VAS, VAD, and VAD+VA diets. NB: Milk data at DPP18–21 for the mock group is missing since the sows ceased lactating after losing all the piglets. Error bars denote SE.

Supplementary Figure 3 Frequencies (%) of activated (Foxp3^-^CD25^+^) **(A, D)**, inducible (Foxp3^+^CD25^-^) **(B, E)**, and natural (Foxp3^+^CD25^+^) **(C, F)** Treg cell subpopulations among the CD4^+^ (upper) and CD8^+^ (lower) T cell populations in the blood of PEDV/mock-exposed sows fed on VAS, VAD, and VAD+VA diets during gestation and lactation period. NB: Milk data at DPP18–21 for the mock group is missing since the sows ceased lactating after losing all the piglets. Error bars denote SE.

Supplementary Figure 4 Frequencies (%) of activated (Foxp3^-^CD25^+^) **(A, D)**, inducible (Foxp3^+^CD25^-^) **(B, E)**, and natural (Foxp3^+^CD25^+^) **(C, F)** Treg cell subpopulations among the CD4^+^ (upper) and CD8^+^ (lower) T cell populations in the colostrum/milk of PEDV/mock-exposed sows fed on VAS, VAD, and VAD+VA diets during gestation and lactation period. NB: Milk data at DPP18–21 for the mock group is missing since the sows ceased lactating after losing all the piglets. Error bars denote SE.

Supplementary Figure 5 Frequencies (%) of activated (Foxp3^-^CD25^+^) **(A, D)**, inducible (Foxp3^+^CD25^-^) **(B, E)**, and natural (Foxp3^+^CD25^+^) **(C, F)** Treg cell subpopulations among the CD4^+^ (upper) and CD8^+^ (lower) T cell populations in various tissues at euthanasia (DPP18–21) of PEDV/mock exposed sows fed on VAS, VAD and VAD+VA diets during gestation and lactation period. MLN, mesenteric lymph nodes; MG, mammary gland. NB: Milk data at DPP18–21 for the mock group is missing since the sows ceased lactating after losing all the piglets. Error bars denote SE.

Supplementary Figure 6 Immunofluorescence of MAdCAM-1-stained MG of VAS-mock sow (sow 2646) **(A, B)** and VAD+VA+PEDV sow (sow 631) **(C, D)** at DPP3–5, showing strong positivity (green, arrows) on the endothelial cells lining the vessels of the MG. ImageJ-processed, MAdCAM-1-positive areas (red, arrows) (right panel). Original magnification, all ×200, scale bars = 50 µm.

Supplementary Figure 7 Immunofluorescence of CCL25-stained MG of VAS+PEDV sow (sow 198) **(A, B)** and VAD+VA-mock sow (sow 98) **(C, D)** at DPP3–5, showing strong positivity (green, arrows) on the endothelial cells lining the vessels of MG. ImageJ-processed, CCL25-positive areas (red, arrows) (right panel). Original magnification, all ×200, scale bars = 50 µm.

Supplementary Figure 8 Immunofluorescence of pIgR-stained MG of VAD-mock sow (sow 5305) **(A, B)** and VAD+VA-mock sow (sow 5296) **(C, D)** at GD110–115, showing strong positivity (green, arrows) on the epithelial cells lining the alveoli of MG. ImageJ-processed, pIgR-positive areas (red, arrows) (right panel). Original magnification, all ×200, scale bars = 50 µm.

Supplementary Figure 9 Mean numbers of IgA^+^ **(A)** and IgG^+^ **(B)** cells and mean areas (%) positive per microscopic area, ×200 for MAdCAM-1 **(C)**, CCL25 **(D)**, and pIgR **(E)** cells in MG of PEDV/mock-inoculated sows fed VAS, VAD, and VAD+VA-supplemented diets during gestation and postpartum. Error bars denote SE.

Supplementary Table 1 Mean frequencies (%) of Ig-secreting (CD2^+^CD21^-^), memory/resting (CD2^-^CD21^-^), activated/primed (CD2^-^CD21^+^), and naïve (CD2^+^CD21^+^) B cells; IgA^+^, α4^+^β7^+^, IgA^+^α4^+^β7^+^, and CCR10^+^ cell subpopulations among CD79β^+^ B lymphocytes in the blood, milk, and selected tissues of PEDV/mock-inoculated sows fed VAS, VAD, and VAD+VA supplemented diets during gestation and postpartum. NB: Milk data at DPP18–21 for the mock group is missing since the sows ceased lactating after losing all the piglets.
