# Supplementary figures and images for: Maternal immunization and vitamin A sufficiency impact sow primary adaptive immunity and passive protection to nursing piglets against porcine epidemic diarrhea virus infection
Source: Front Immunol. 2024 May 15;15:1397118. doi: 10.3389/fimmu.2024.1397118 (PMC11133611; doi:10.3389/fimmu.2024.1397118)

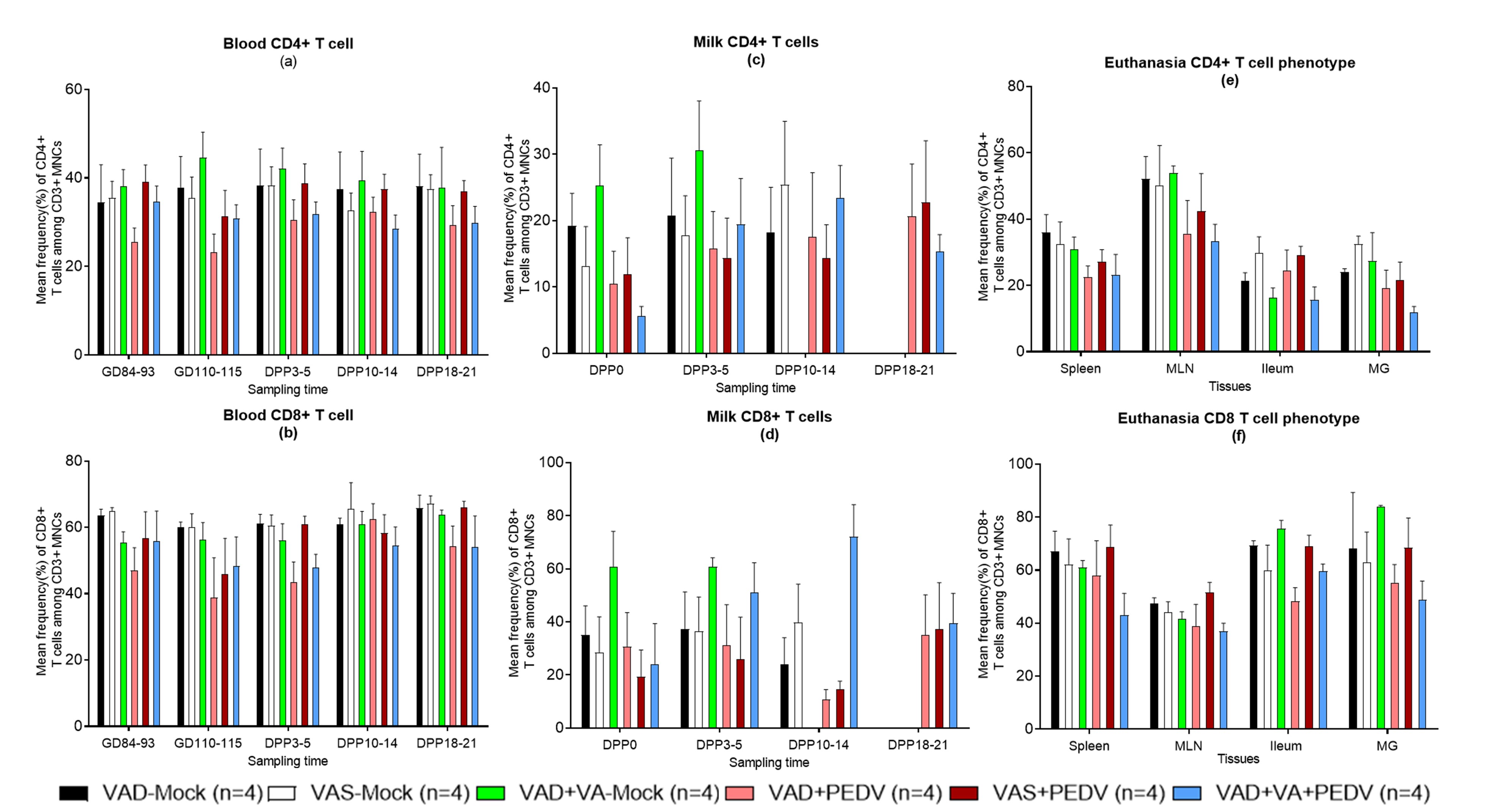

Supplement: Supplementary Figure 1 — Frequencies (%) of CD3+CD4+ (A, C, E) and CD3+CD8+ (B, D, F) cells in the blood, milk, and tissues of PEDV/mock-exposed sows fed VAS, VAD, and VAD+VA diets. MLN, mesenteric lymph nodes; MG, mammary gland. NB: Milk data at DPP18–21 for the mock group is missing since the sows ceased lactating after losing all the piglets. Error bars denote SE. [file Image_1.jpeg]

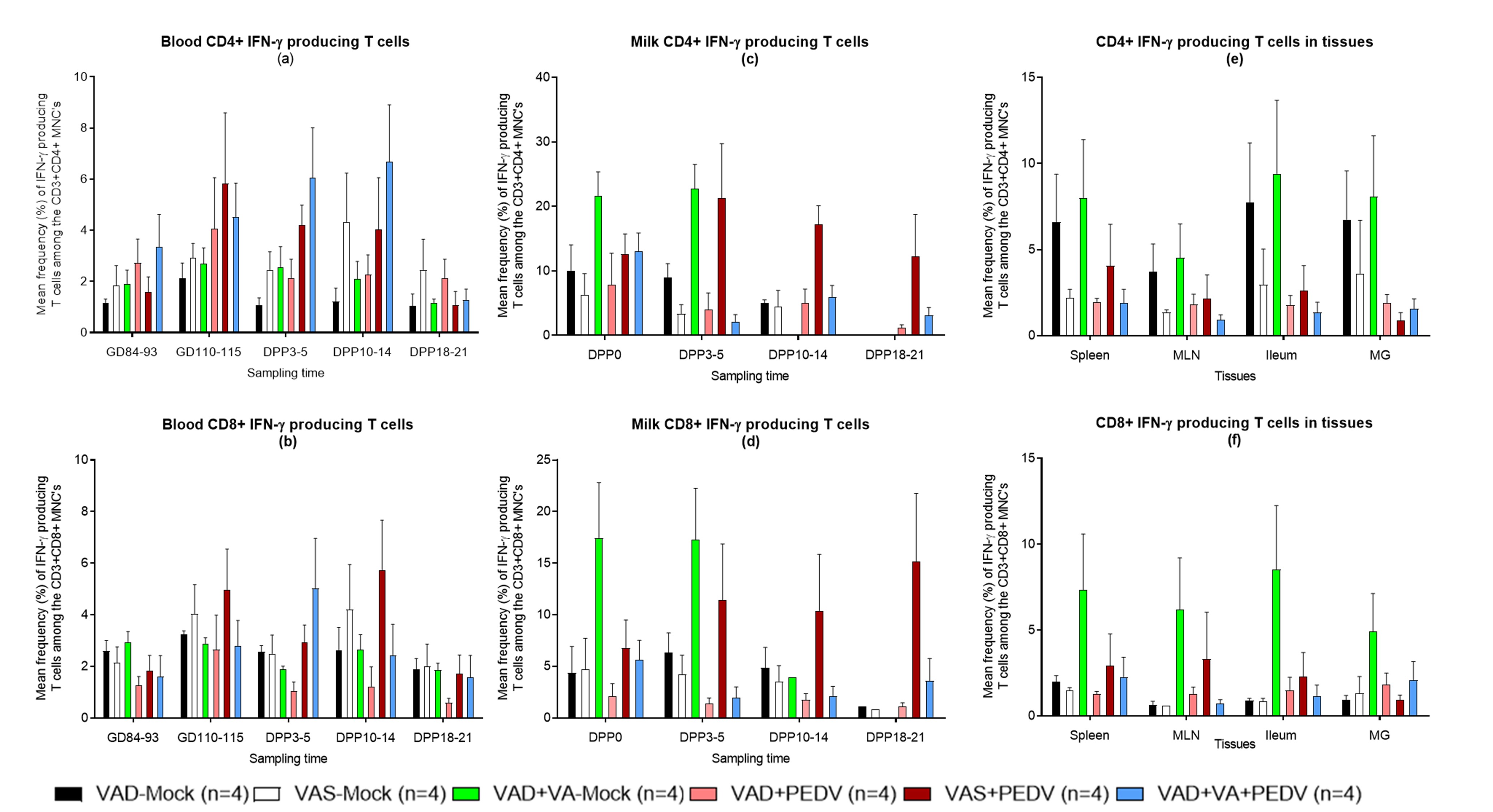

Supplement: Supplementary Figure 2 — Frequencies (%) of IFN-γ producing cells among the CD3+CD4+ (A, C, E) and CD3+CD8+ (B, D, F) T cells in the blood, milk, and tissues of PEDV/mock-exposed sows fed VAS, VAD, and VAD+VA diets. NB: Milk data at DPP18–21 for the mock group is missing since the sows ceased lactating after losing all the piglets. Error bars denote SE. [file Image_2.jpeg]

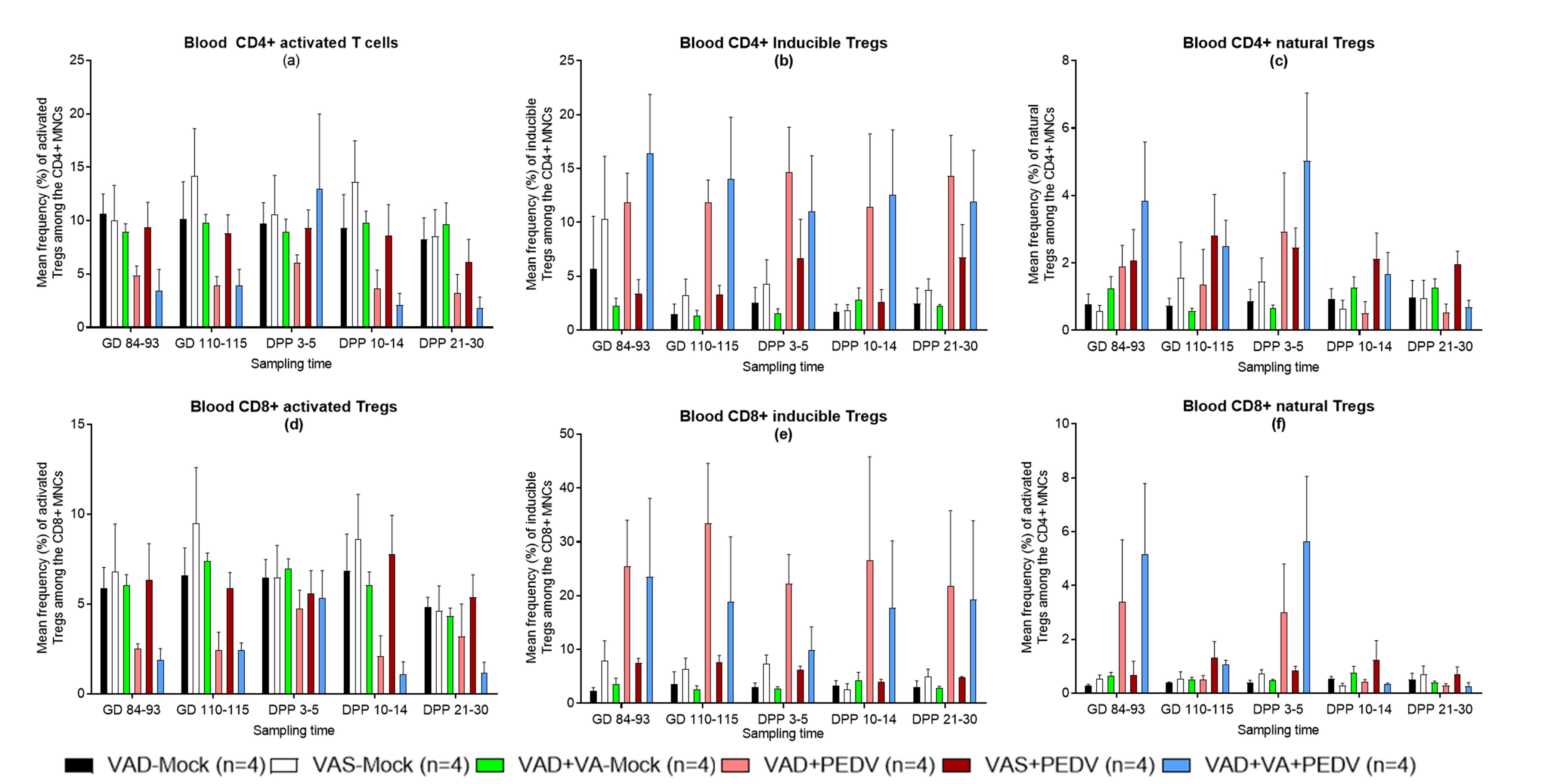

Supplement: Supplementary Figure 3 — Frequencies (%) of activated (Foxp3-CD25+) (A, D), inducible (Foxp3+CD25-) (B, E), and natural (Foxp3+CD25+) (C, F) Treg cell subpopulations among the CD4+ (upper) and CD8+ (lower) T cell populations in the blood of PEDV/mock-exposed sows fed on VAS, VAD, and VAD+VA diets during gestation and lactation period. NB: Milk data at DPP18–21 for the mock group is missing since the sows ceased lactating after losing all the piglets. Error bars denote SE. [file Image_3.jpeg]

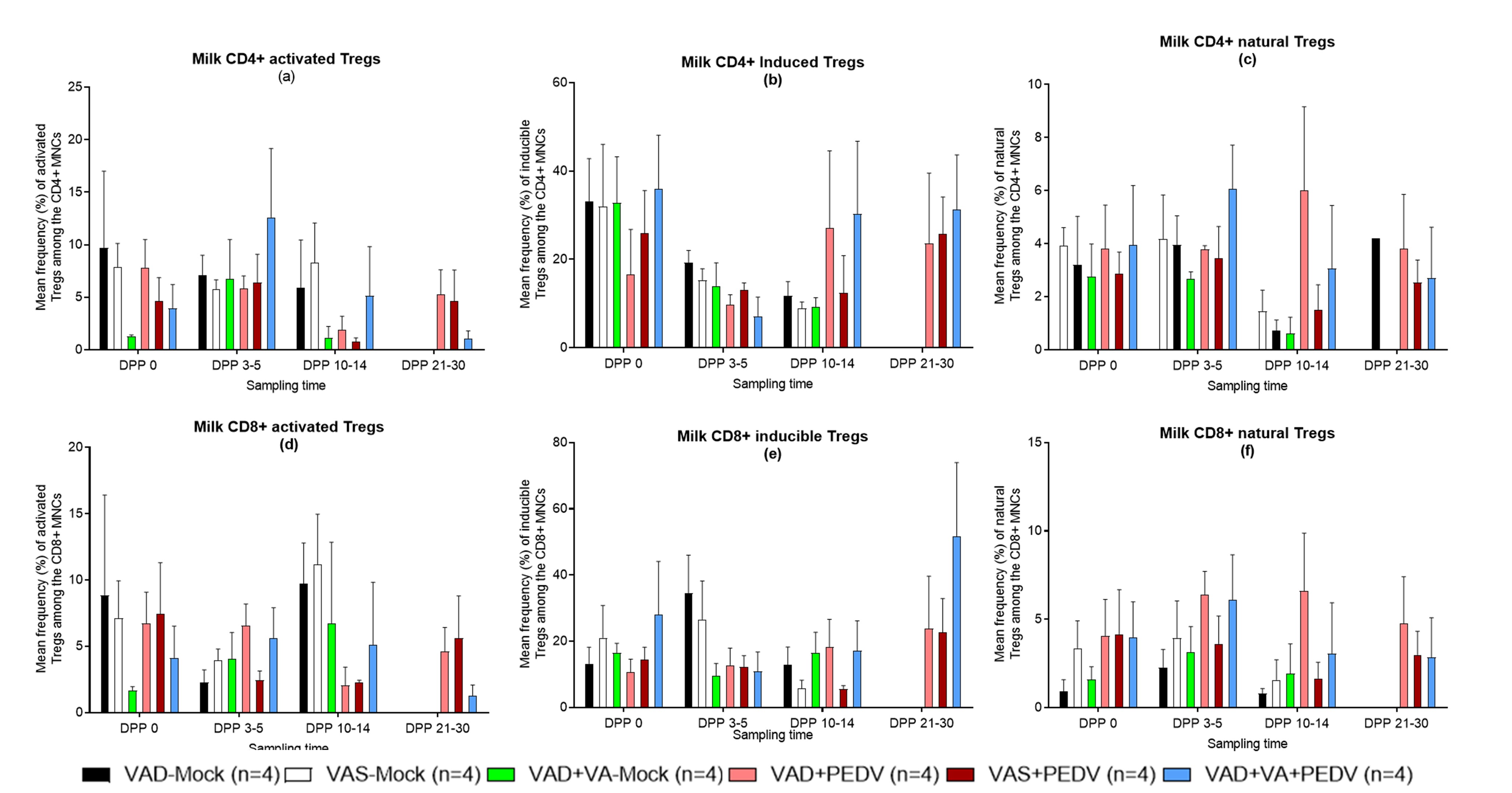

Supplement: Supplementary Figure 4 — Frequencies (%) of activated (Foxp3-CD25+) (A, D), inducible (Foxp3+CD25-) (B, E), and natural (Foxp3+CD25+) (C, F) Treg cell subpopulations among the CD4+ (upper) and CD8+ (lower) T cell populations in the colostrum/milk of PEDV/mock-exposed sows fed on VAS, VAD, and VAD+VA diets during gestation and lactation period. NB: Milk data at DPP18–21 for the mock group is missing since the sows ceased lactating after losing all the piglets. Error bars denote SE. [file Image_4.jpeg]

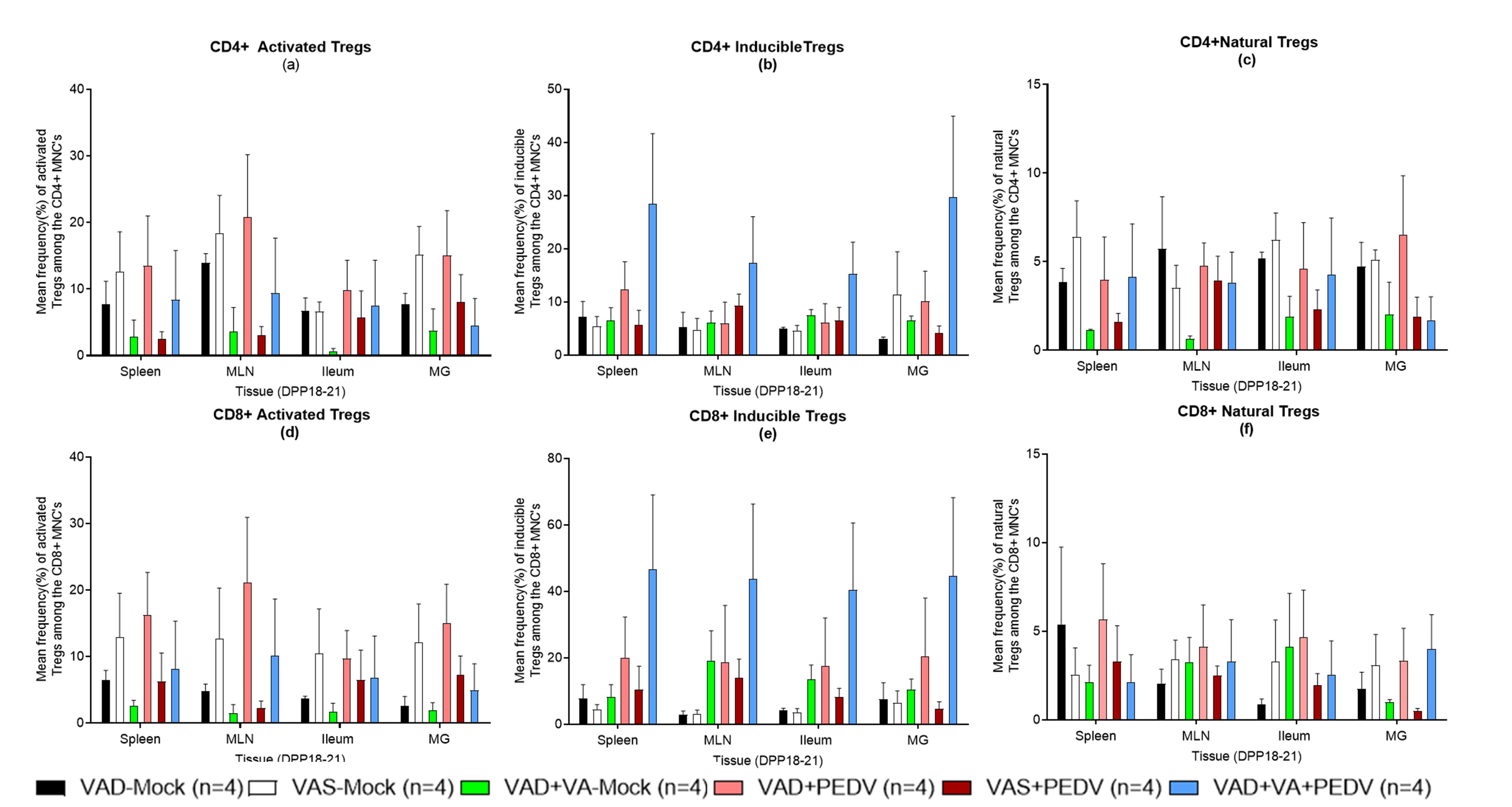

Supplement: Supplementary Figure 5 — Frequencies (%) of activated (Foxp3-CD25+) (A, D), inducible (Foxp3+CD25-) (B, E), and natural (Foxp3+CD25+) (C, F) Treg cell subpopulations among the CD4+ (upper) and CD8+ (lower) T cell populations in various tissues at euthanasia (DPP18–21) of PEDV/mock exposed sows fed on VAS, VAD and VAD+VA diets during gestation and lactation period. MLN, mesenteric lymph nodes; MG, mammary gland. NB: Milk data at DPP18–21 for the mock group is missing since the sows ceased lactating after losing all the piglets. Error bars denote SE. [file Image_5.jpeg]

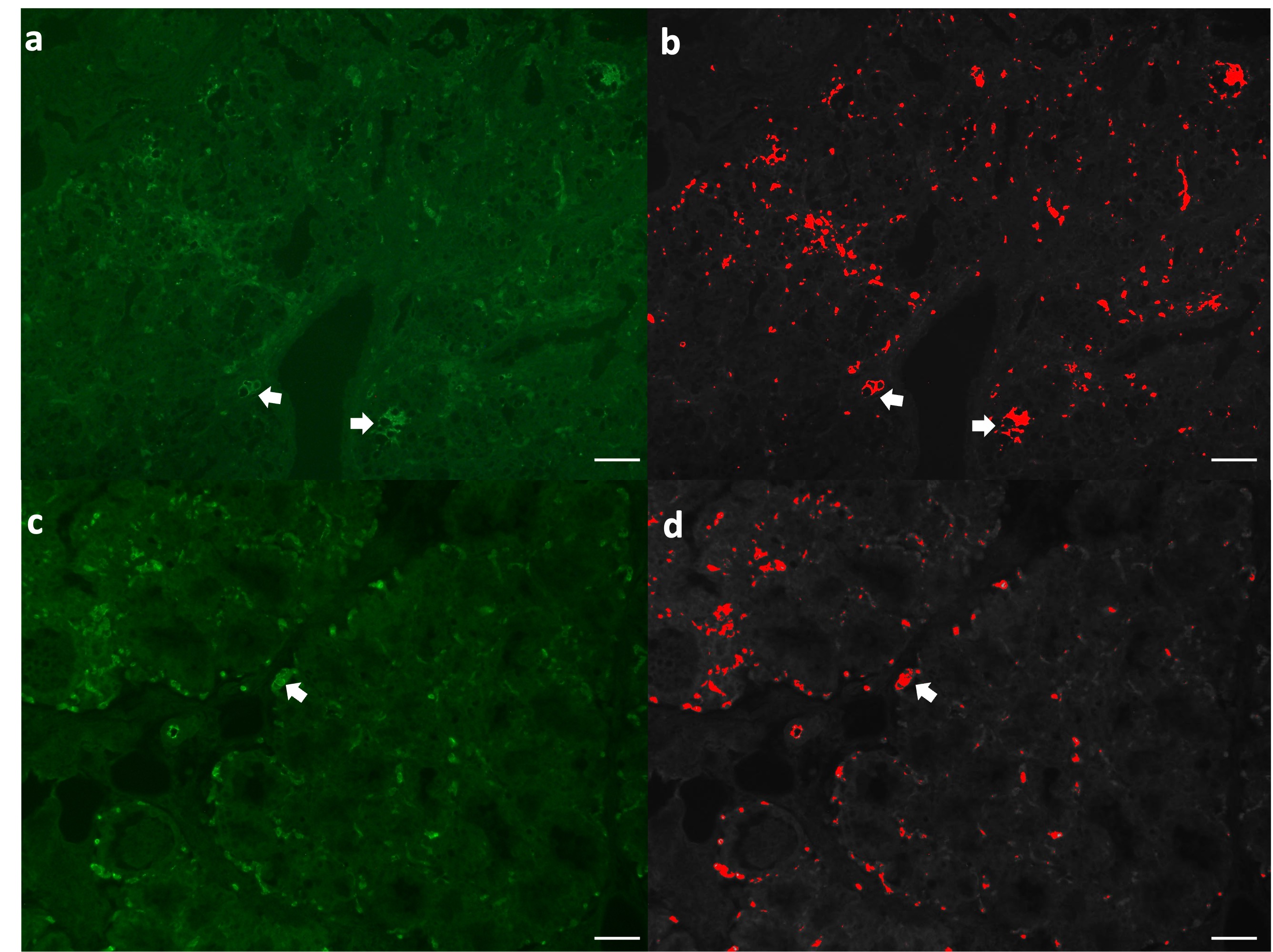

Supplement: Supplementary Figure 6 — Immunofluorescence of MAdCAM-1-stained MG of VAS-mock sow (sow 2646) (A, B) and VAD+VA+PEDV sow (sow 631) (C, D) at DPP3–5, showing strong positivity (green, arrows) on the endothelial cells lining the vessels of the MG. ImageJ-processed, MAdCAM-1-positive areas (red, arrows) (right panel). Original magnification, all ×200, scale bars = 50 µm. [file Image_6.jpeg]

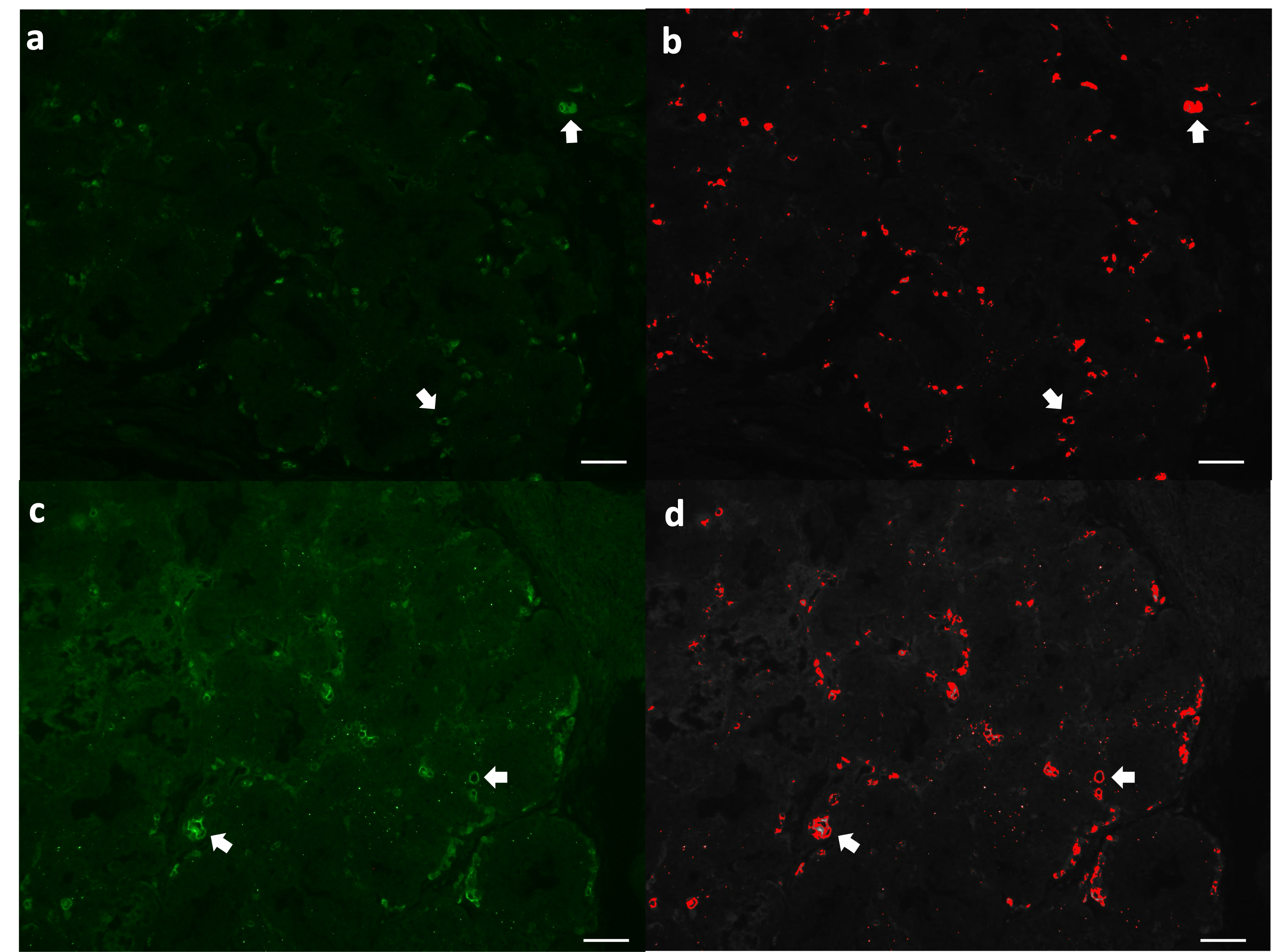

Supplement: Supplementary Figure 7 — Immunofluorescence of CCL25-stained MG of VAS+PEDV sow (sow 198) (A, B) and VAD+VA-mock sow (sow 98) (C, D) at DPP3–5, showing strong positivity (green, arrows) on the endothelial cells lining the vessels of MG. ImageJ-processed, CCL25-positive areas (red, arrows) (right panel). Original magnification, all ×200, scale bars = 50 µm. [file Image_7.png]

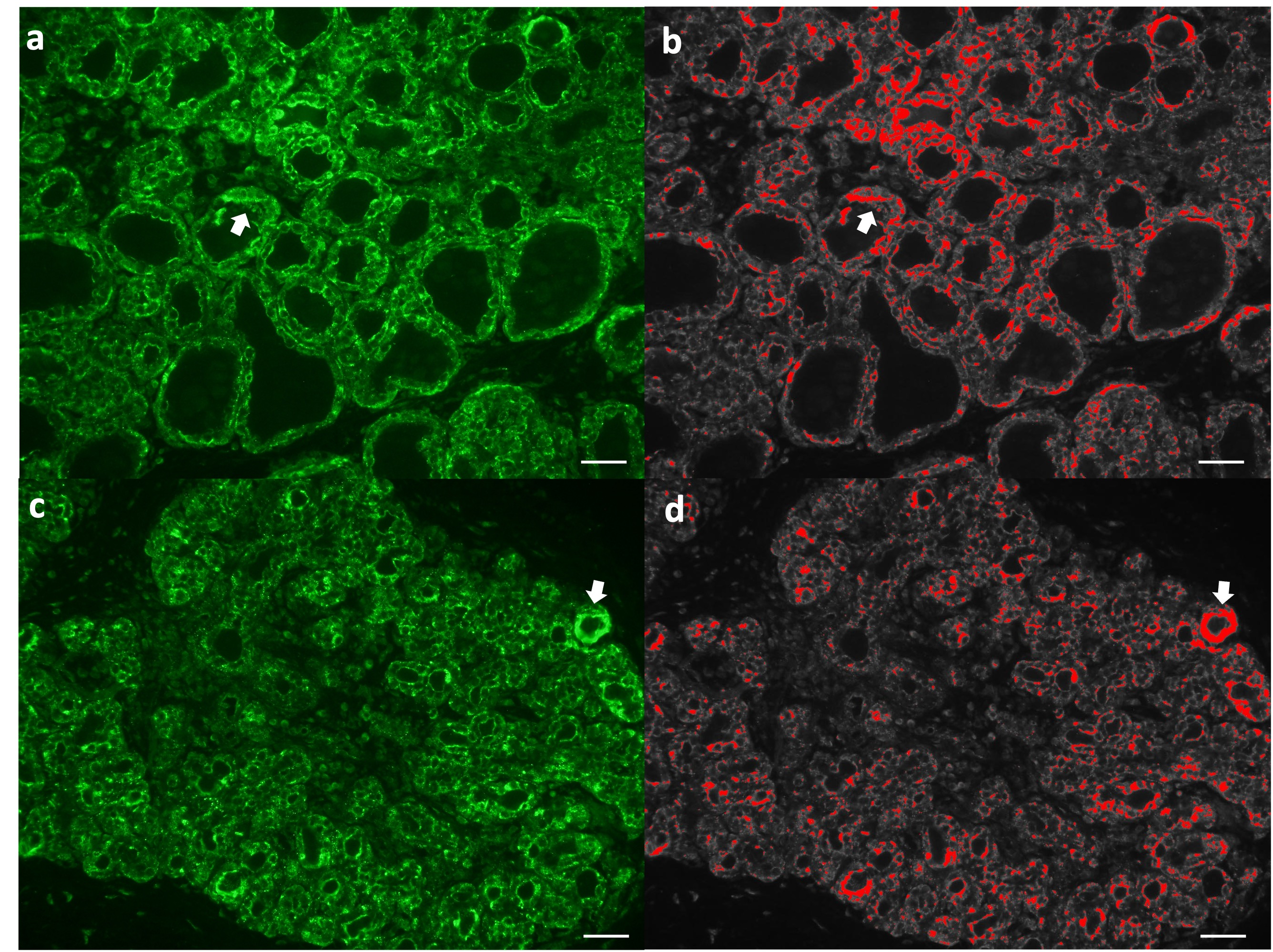

Supplement: Supplementary Figure 8 — Immunofluorescence of pIgR-stained MG of VAD-mock sow (sow 5305) (A, B) and VAD+VA-mock sow (sow 5296) (C, D) at GD110–115, showing strong positivity (green, arrows) on the epithelial cells lining the alveoli of MG. ImageJ-processed, pIgR-positive areas (red, arrows) (right panel). Original magnification, all ×200, scale bars = 50 µm. [file Image_8.jpeg]

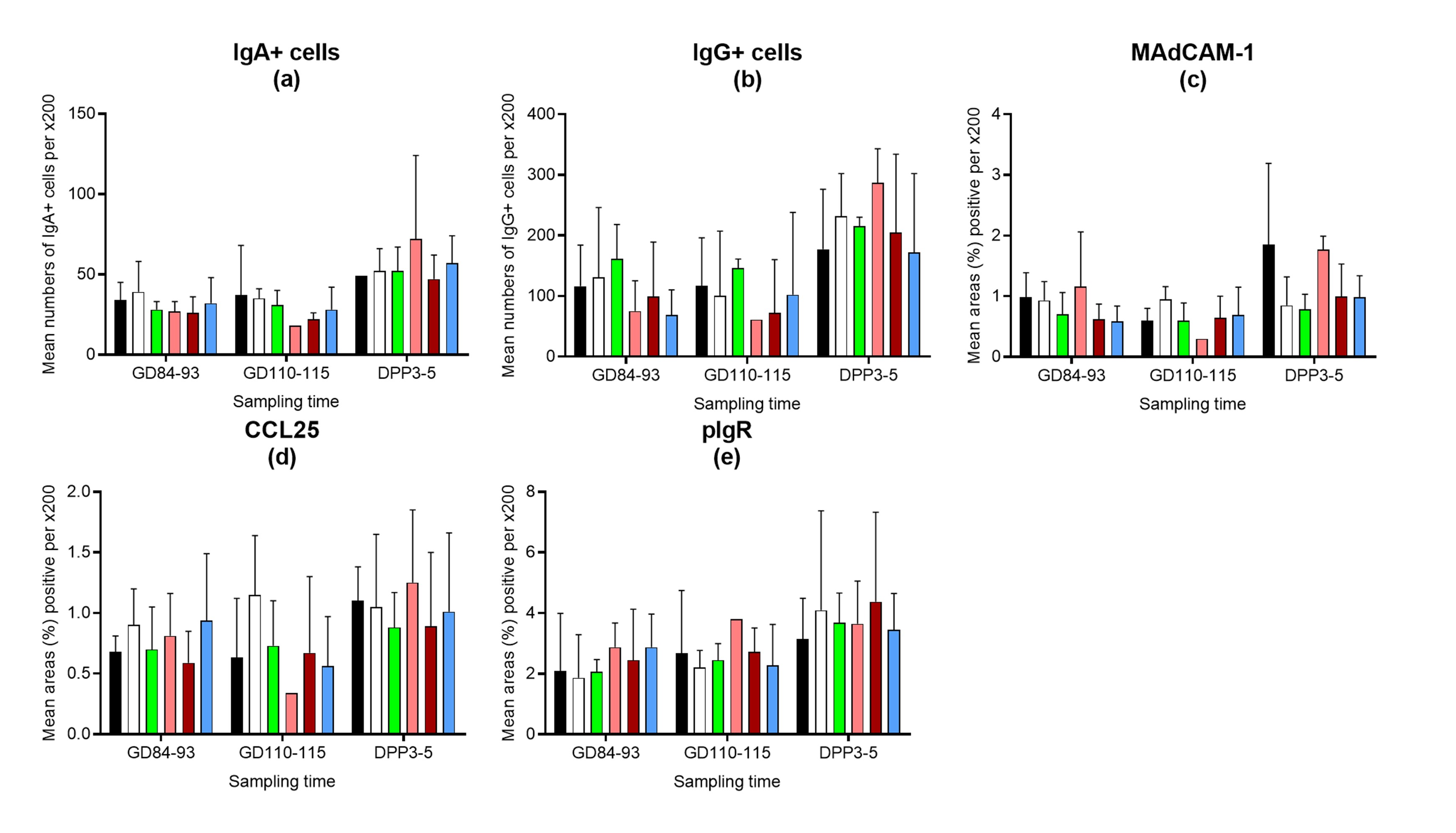

Supplement: Supplementary Figure 9 — Mean numbers of IgA+ (A) and IgG+ (B) cells and mean areas (%) positive per microscopic area, ×200 for MAdCAM-1 (C), CCL25 (D), and pIgR (E) cells in MG of PEDV/mock-inoculated sows fed VAS, VAD, and VAD+VA-supplemented diets during gestation and postpartum. Error bars denote SE. [file Image_9.jpeg]
